# Development and validation of a high-confidence diagnostic model integrating ctDNA methylation and serum biomarkers for early-stage hepatocellular carcinoma detection

**DOI:** 10.1186/s43556-026-00426-3

**Published:** 2026-03-11

**Authors:** Han Wu, Mingda Wang, Zhiyi Wan, Lanqing Yao, Shuang Zhou, Hui Wang, Guoyue Lv, Nanya Wang, Fengmei Wang, Jiahao Xu, Xinfei Xu, Chao Li, Yongkang Diao, Timohty M. Pawlik, Rui Liu, Feng Shen, Tian Yang

**Affiliations:** 1 Department of Hepatobiliary Surgery, Eastern Hepatobiliary Surgery Hospital, Naval Medical University, Shanghai, China; 2 Singlera Genomics (Shanghai) Inc., Shanghai, China; 3 Department of Hepatobiliary and Pancreatic Surgery, General Surgery Center, First Hospital of Jilin University, Changchun, Jilin, China; 4 Phase I Clinical Trials Unit, First Hospital of Jilin University, Changchun, Jilin, China; 5 Department of Gastroenterology and Hepatology, Tianjin Third Central Hospital, Tianjin, China; 6 Department of Surgery, Ohio State University, Wexner Medical Center, Columbus, OH USA

**Keywords:** Hepatocellular carcinoma, Early detection, CtDNA methylation, GAMAD, At-risk population

## Abstract

Hepatocellular carcinoma (HCC) remains a leading cause of cancer-related mortality, with significantly improved prognosis when detected early in high-risk populations. Current serum biomarkers show limited sensitivity for early-stage disease. We developed and validated GAMAD, a multimodal diagnostic model integrating circulating tumor DNA (ctDNA) methylation with established serum markers to enhance early HCC detection in China. In this multicenter prospective trial, a total of 1,692 patients were enrolled: 476 with HCC, 645 with hepatitis, 443 with cirrhosis, and 128 with no detectable liver abnormalities. Blood tests including AFP, AFP-L3, DCP, and ctDNA methylation (HepaAiQ) were performed. Using training, validation, and independent test cohorts, we developed the GAMAD model by integrating HepaAiQ with gender, age, AFP, and DCP. HepaAiQ demonstrated a superior performance compared with AFP, DCP, and AFP-L3 with a sensitivity of 74.6%, specificity of 88.1%, and an area under the curve (AUC) of 0.862 (95% CI, 0.842–0.883) in all samples. The GAMAD model, optimized specifically for early-stage HCC, achieved sensitivity of 80.5%, specificity of 90.4%, and AUC of 0.934 (95% CI, 0.911–0.957) in the validation cohort. In the independent test cohort, GAMAD showed superior performance with sensitivity of 86.5% for stage 0/A and 91.7% for stage B-C HCC (AUC 0.952, 95% CI, 0.931–0.973), substantially outperforming the GALAD model across all cohorts. The GAMAD model represents a significant clinical advancement for early HCC detection. Its intended clinical use is to serve as an adjunct to standard imaging for high-risk populations, substantially increasing early diagnosis rates.

**Supplementary Information:**

The online version contains supplementary material available at 10.1186/s43556-026-00426-3.

## Introduction

Hepatocellular carcinoma (HCC) accounts for the majority of primary liver cancers and remains a leading cause of cancer-related mortality worldwide [1]. The five-year survival rate for HCC patients in China is only 14.4%, markedly lower than the national average for all cancers (43.7%) [2]. This poor prognosis is largely attributable to insufficient early detection among high-risk populations, most commonly those with chronic hepatitis B virus (HBV) infection, hepatitis C virus infection, or cirrhosis. More than 80% of HCC cases in China are attributable to hepatitis virus infection [3, 4], underscoring the urgent need for improved surveillance strategies.

Current guidelines recommend that patients with cirrhosis or hepatitis undergo semiannual abdominal ultrasonography (US) with or without serum alpha‑fetoprotein (AFP) measurement for HCC surveillance [5, 6]. However, US has limited sensitivity in patients with fibrosis or fatty liver, and its accuracy is operator dependent. Serum tumor marker assays—such as AFP, the Lens culinaris agglutinin-reactive fraction of AFP (AFP‑L3), and des‑γ‑carboxyprothrombin (DCP)—are widely used but have limited diagnostic accuracy when used alone [7, 8]. These limitations highlight the need for more reliable, noninvasive tools to enhance early detection.

Liquid biopsy approaches, particularly circulating tumor DNA (ctDNA) methylation profiling, have emerged as promising blood-based tools for the early diagnosis of HCC [9–11]. Methylation changes in ctDNA can identify HCC more effectively than traditional US or serum tumor markers [12, 13]. Recently, a noninvasive, quantitative methylation-specific PCR assay (HepaAiQ) was developed to quantify multiple HCC-specific differentially methylated regions. This method demonstrates potential in the diagnosis, early detection, and prognosis of HCC, offering substantial benefits to at-risk populations [14].

Given the etiological and biological heterogeneity of HCC, integrating molecular markers with clinical variables may further improve diagnostic performance [15, 16]. The GALAD model, which combines serum markers (AFP, AFP-L3, and DCP) with demographic factors like gender and age, represents an example of this strategy [15].

In this study, we aimed to identify the most significant demographic, serological, and molecular risk factors for early-stage HCC and to develop a diagnostic model tailored for high-risk patients in China. We hypothesized that integrating HepaAiQ methylation scores with conventional tumor markers and clinical variables would improve diagnostic accuracy compared with existing strategies. Using a large, well-characterized cohort, we validated this model and demonstrated its potential to enhance early detection.

## Results

### Study design and patient’s clinical characteristics

**Fig. 1:**
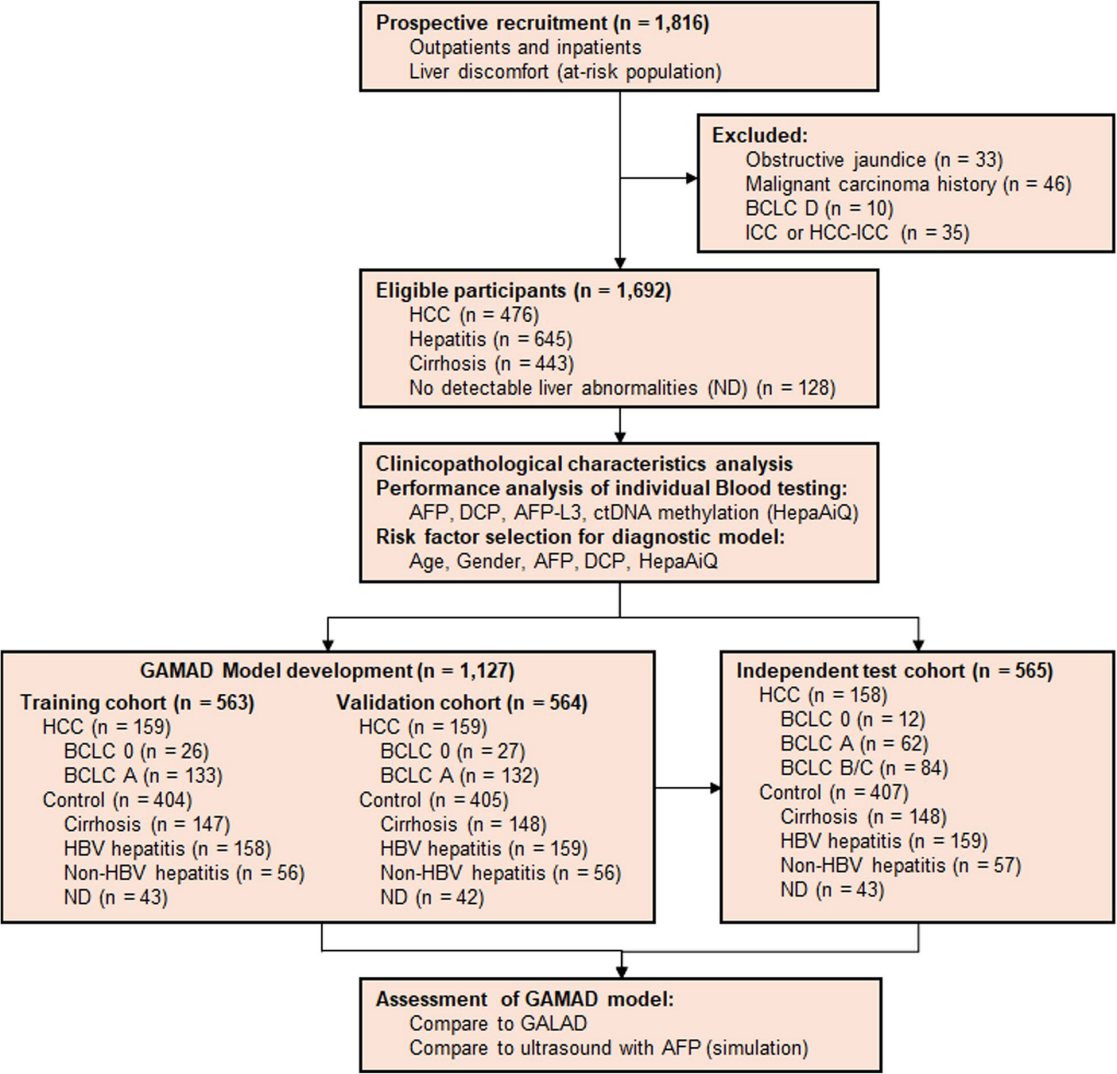
Schematic diagram of the research design. The study consisted of three main phases: identification of individual biomarker performance, development andvalidation of a multifactorial diagnostic model, and assessment of the multifactorial diagnostic model. AFP: alpha-fetoprotein; BCLC: Barcelona Clinic Liver Cancer; DCP: des-gamma-carboxy prothrombin; HBV: hepatitis B virus; HCC: hepatocellular carcinoma; HCC-ICC: combined hepatocellular and intrahepatic cholangiocarcinoma; ND: patients with no detectable liver abnormalities

The study prospectively recruited 1,816 patients from three hospitals who were at-risk for liver dysfunction or HCC (Fig. 1). During recruitment, blood samples were collected for testing of AFP, AFP-L3, DCP, and HepaAiQ. After clinical diagnosis was confirmed on pathology, 1,692 individuals were ultimately included in the analytic cohort, comprising 476 patients with HCC and 1,216 controls. The clinical characteristics of the HCC group and the control group are summarized in Table S1. Among patients with HCC, 82.4% (392/476) had BCLC stage 0/A disease, while 17.6% (84/476) were stage BCLC B/C. The control group included 645 (53.0%) patients with hepatitis, 443 (36.4%) with cirrhosis, and 128 (10.5%) patients with ND (Fig. 1, Table S1).

After evaluating individual risk factors, the best-performing factors were selected to construct a multi-variable diagnostic model. Approximately 66.6% (1,127) of patients were used for model development, while the remaining 33.4% (565) of patients were left for independent testing. During model building, the 1,127 patients were evenly divided into training and validation cohorts; HCC patients with stage 0-A were specifically selected to build a model for HCC early detection (Fig. 1, Table S2). The HCC patients in the independent test cohort included all stages. The proportions of different control groups are consistent across the three cohorts, as summarized in Table S3. This design ensured a balanced cohort, allowing the model to be evaluated robustly for both early detection and overall diagnostic performance.

### Performance of HepaAiQ and serum tumor markers

**Fig. 2:**
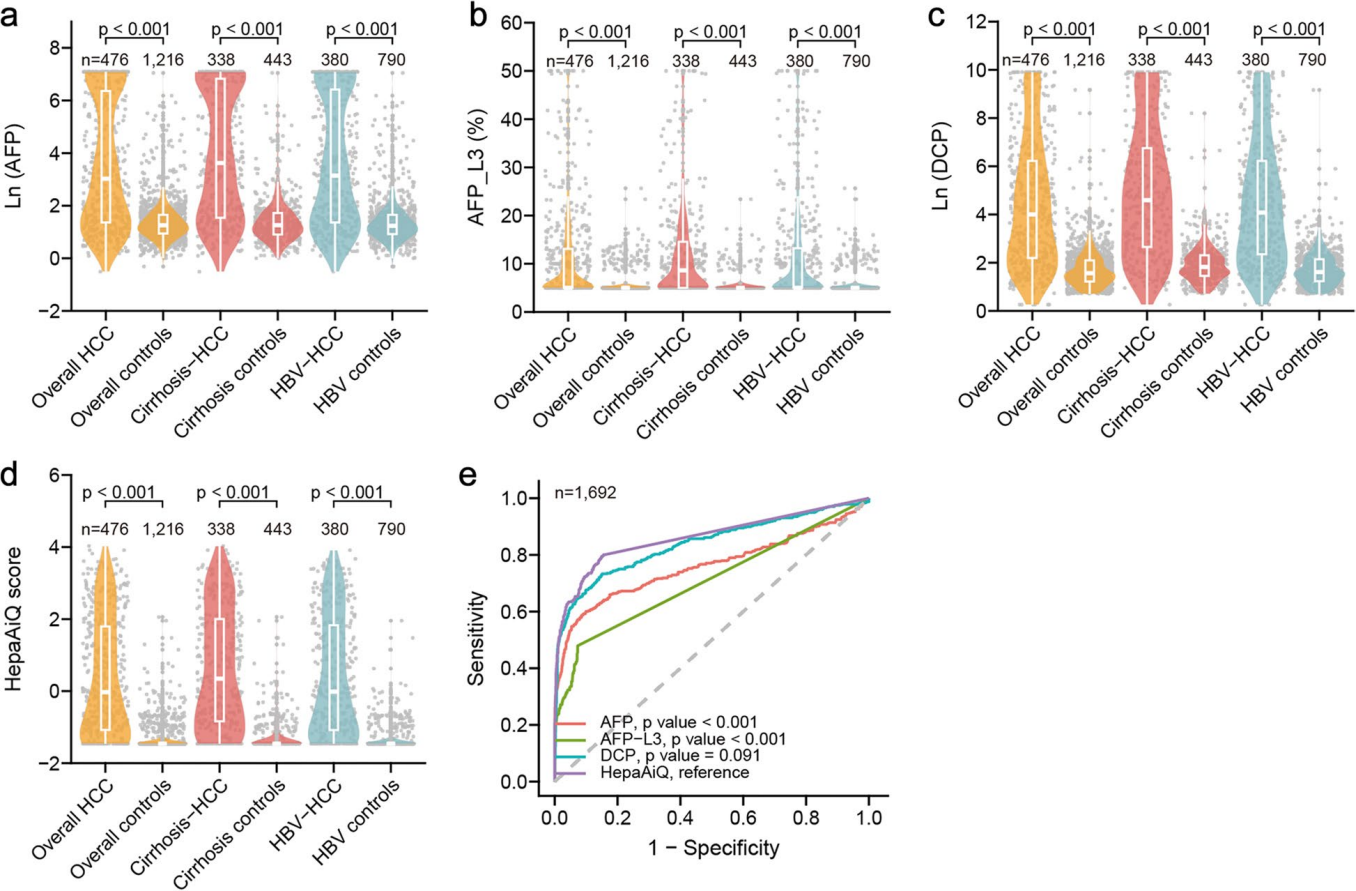
Performance of serum tumor markers and HepaAiQ. Comparisons of Ln (AFP) (**a**), AFP-L3 (**b**), Ln (DCP) (**c**), and HepaAiQ score (**d**) between HCC cases and respective controls. Mann–Whitney U-test. **e** Comparisons of ROC curves for the detection of HCC among AFP, AFP-L3, DCP, and HepaAiQ. DeLong's test. AFP: alpha-fetoprotein; BCLC: Barcelona Clinic Liver Cancer; DCP: des-gamma-carboxy prothrombin; HBV: hepatitis B virus; HCC: hepatocellular carcinoma

We then analyzed the diagnostic performance of HepaAiQ and serum tumor markers for HCC. Although conventional biochemistry parameters were measured, they were not included in the final model due to their limited diagnostic value in detecting HCC (Fig. S1). Building on prior research [14], we optimized the HepaAIQ assay using LASSO regression analysis to screen seven key methylation markers from a candidate pool of 20: *IKZF1*, *Septin9*, *Septin9_region2*, *B4GALNT1*, *BEST4*, *BEND4*, and *GRASP* (Fig. S2). The HepaAiQ assay demonstrated high reliability and accuracy, with intra-assay coefficient of variation (CV) of Ct values ranging from 0% to 0.73% and inter-assay CV from 0% to 2.61% across three reagent lots and three gradient methylated DNA samples (Table S4). The diagnostic efficacy of several blood markers, including AFP, DCP, AFP-L3, and ctDNA methylation via HepaAiQ was initially assessed in all samples. The values of Ln (AFP), AFP-L3, Ln (DCP), and HepaAiQ scores in HCC were significantly higher than the corresponding controls (Fig. 2a-d). Additionally, HepaAiQ demonstrated a superior overall performance (74.6% sensitivity, 88.1% specificity) than AFP (50.6% sensitivity, 95.8% specificity), AFP-L3 (37.8% sensitivity, 94.2% specificity) and DCP (53.4% sensitivity, 97.6% specificity) (Table S5). Importantly, the sensitivity of HepaAiQ for BCLC stage 0-A HCC was 71.2%, which was markedly higher than AFP (45.4%), AFP-L3 (31.6%), and DCP (47.2%). The AUC for HepaAiQ, AFP, AFP-L3, and DCP was 0.862 (95% confidence interval (CI), 0.842–0.883, reference), 0.761 (95% CI, 0.73–0.791, *P* < 0.001), 0.708 (95% CI, 0.684–0.732, *P* < 0.001) and 0.839 (95% CI, 0.814–0.863, *P* < 0.001), respectively (Fig. 2e).

The performance of HepaAiQ was subsequently evaluated in samples that were negative for the other markers. HepaAiQ reduced the number of false-negative HCC samples by 154 (65.5%, 154/235) versus AFP, 131 (59%, 131/222) compared to DCP, 200 (67.6%, 200/296) compared to AFP-L3, 86 (56.2%, 86/153) compared to AFP/DCP, and 86 (56.2%, 86/153) compared to AFP/DCP/AFP-L3 (Table S6). Furthermore, HepaAiQ also reduced the number of false positives in the control samples by 38 (74.5%, 38/51) with AFP, 18 (62.1%, 18/29) with DCP, 56 (80%, 56/70) with AFP-L3, 52 (70.3%, 52/74) with AFP/DCP, and 75 (72.8%, 75/103) with AFP/DCP/AFP-L3 (Table S6). HepaAiQ outperformed conventional serum markers, underscoring its potential as a sensitive and specific diagnostic tool.

### Building of an early HCC diagnostic model GAMAD

**Fig. 3:**
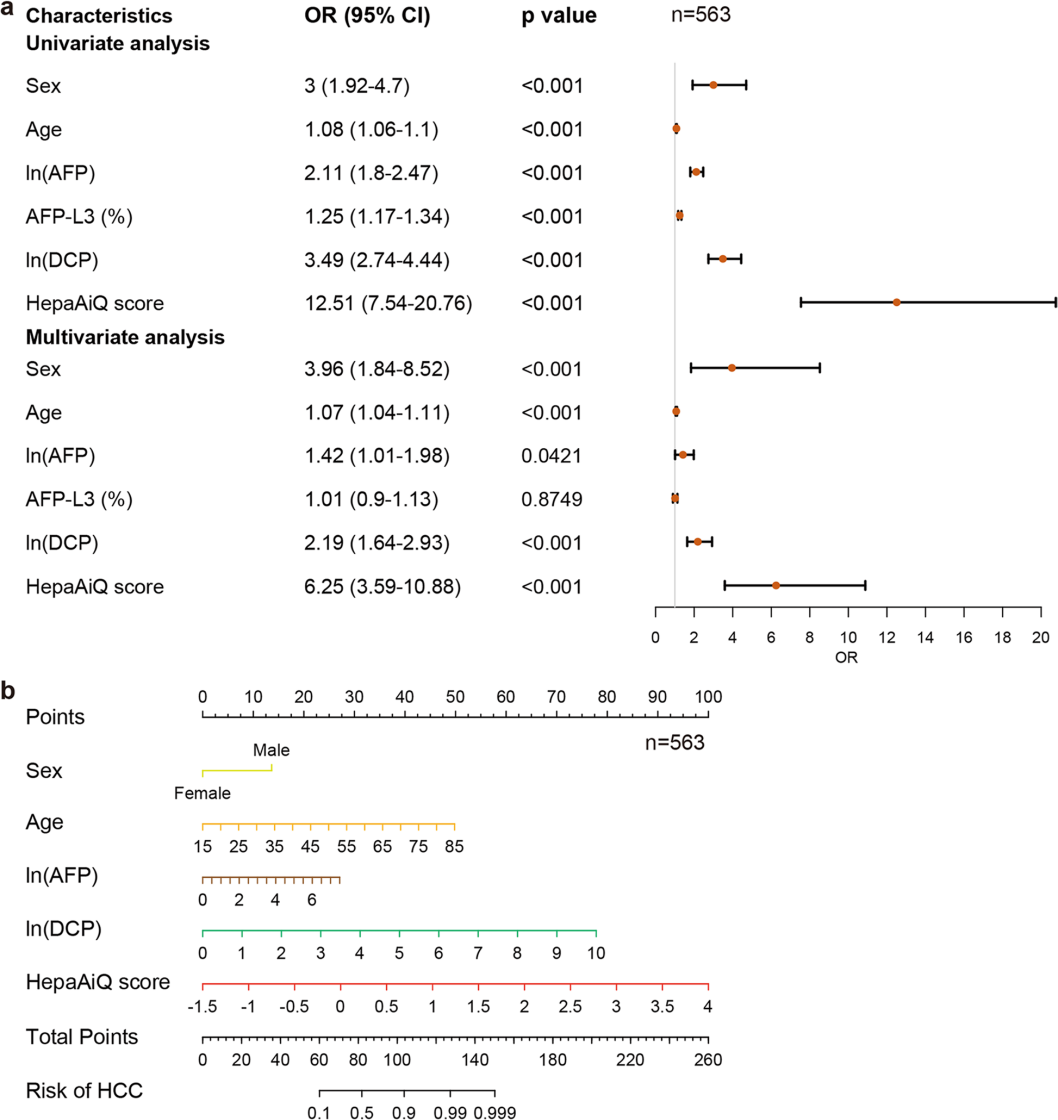
Development of a diagnostic model "GAMAD" for early HCC. **a** Univariate and multivariate regression analyses of the independent risk factors associated with the incidence of HCC in the training set. OR represented the odds ratio, and 95% CI represented 95% confidence intervals. Wald test. **b** Nomogram to predict early HCC. AFP: alpha-fetoprotein; DCP: des-gamma-carboxy prothrombin; HCC: hepatocellular carcinoma

We then hypothesized that combining multiple features might improve the diagnostic accuracy of HCC in at-risk populations. To train a multi-factor model, we specifically identified BCLC stage 0-A HCC patients in the training cohort. Univariate and multivariate logistic regression analyses were used to incorporate basic, clinical, and testing markers in the training group. The analysis demonstrated that all variables, except for AFP-L3, were correlated with the incidence of HCC (Fig. 3a and Table S7). AFP-L3 had a lower diagnostic accuracy compared with AFP or DCP and was not significant in the multivariate models.

**Fig. 4:**
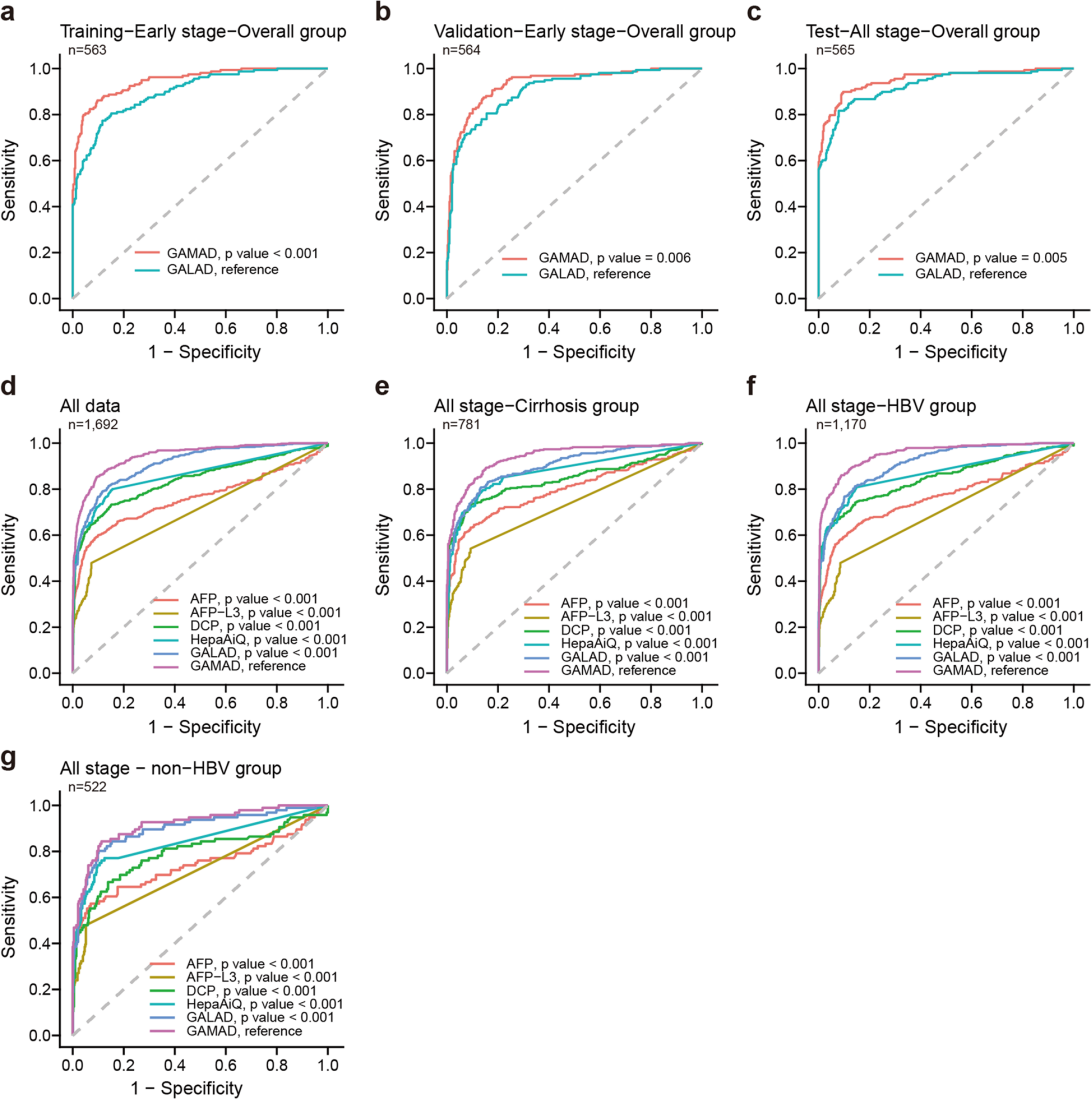
GAMAD model performance assessment. Comparisons of ROC curves for the detection of HCC among GAMAD model and GALAD model in (**a**) training cohort, (**b**) validation cohort, and (**c**) test cohort. Comparisons of ROC curves for the detection of HCC among individual markers, GAMAD model, and GALAD model in the overall group (**d**), cirrhosis subgroup (**e**), HBV subgroup (**f**), and non-HBV subgroup (**g**). DeLong's test. AFP: alpha-fetoprotein; DCP: des-gamma-carboxy prothrombin; HBV: hepatitis B virus; HCC: hepatocellular carcinoma

Subsequently, we utilized *G*ender, *A*ge, DNA *M*ethylation (HepaAiQ score), *A*FP, and *D*CP as variables to develop a nomogram-based calculator in the training cohort, designated as the GAMAD model (Fig. 3b). It was optimized to ensure a specificity greater than 90%, with the cutoff value chosen to provide the highest sensitivity. The resultant AUC for the GAMAD model in the training cohort was 0.948 (95% CI, 0.928–0.968), achieving a sensitivity of 86.2% for early-stage HCC and a specificity of 90.3%. The model was further validated in the validation set, in which the proportions of HCC, hepatitis, and cirrhosis were consistent with the training set. The performance of GAMAD was highly reproducible with an AUC of 0.934 (95% CI, 0.911–0.957), corresponding to a sensitivity of 80.5% in early-stage HCC and a specificity of 90.4% (Table 1 and Fig. 4a-b). In summary, the GAMAD model demonstrated robust diagnostic accuracy and reproducibility for early-stage HCC detection across training and validation cohorts.

**Table 1:** Diagnostic performances of GAMAD in training, validation, and test cohort

Sample type	Positive	Negative	Total	Sensitivity (95% CI)	Specificity (95% CI)
**Training**					
**HCC**	137	22	159	86.2% (79.9%−90.7%)	
BCLC 0-A	137	22	159	86.2% (79.9%−90.7%)	
BCLC 0	17	9	26	65.4% (46.2%−80.6%)	
BCLC A	120	13	133	90.2% (84%−94.2%)	
BCLC B-C	0	0	0		
**Control**	39	365	404		90.3% (87.1%−92.9%)
Cirrhosis	23	124	147		84.4% (77.6%−89.3%)
HBV Hepatitis	11	147	158		93% (88%−96.1%)
Non-HBV Hepatitis	4	52	56		92.9% (83%−97.2%)
ND	1	42	43		97.7% (87.9%−99.6%)
**Validation**					
**HCC**	128	31	159	80.5% (73.7%−85.9%)	
BCLC 0-A	128	31	159	80.5% (73.7%−85.9%)	
BCLC 0	17	10	27	63% (44.2%−78.5%)	
BCLC A	111	21	132	84.1% (76.9%−89.4%)	
BCLC B-C	0	0	0		
**Control**	39	366	405		90.4% (87.1%−92.9%)
Cirrhosis	22	126	148		85.1% (78.5%−90%)
HBV Hepatitis	11	148	159		93.1% (88%−96.1%)
Non-HBV Hepatitis	3	53	56		94.6% (85.4%−98.2%)
ND	3	39	42		92.9% (81%−97.5%)
**Test**					
**HCC**	141	17	158	89.2% (83.4%−93.2%)	
BCLC 0-A	64	10	74	86.5% (76.9%−92.5%)	
BCLC 0	9	3	12	75% (46.8%−91.1%)	
BCLC A	55	7	62	88.7% (78.5%−94.4%)	
BCLC B-C	77	7	84	91.7% (83.8%−95.9%)	
**Control**	39	368	407		90.4% (87.2%−92.9%)
Cirrhosis	24	124	148		83.8% (77%−88.9%)
HBV Hepatitis	7	152	159		95.6% (91.2%−97.9%)
Non-HBV Hepatitis	6	51	57		89.5% (78.9%−95.1%)
ND	2	41	43		95.3% (84.5%−98.7%)
**Total**					
**HCC**	406	70	476	85.3% (81.8%−88.2%)	
BCLC 0-A	329	63	392	83.9% (80%−87.2%)	
BCLC 0	43	22	65	66.2% (54%−76.5%)	
BCLC A	286	41	327	87.5% (83.4%−90.6%)	
BCLC B-C	77	7	84	91.7% (83.8%−95.9%)	
**Control**	117	1,099	1,216		90.4% (88.6%−91.9%)
Cirrhosis	69	374	443		84.4% (80.8%−87.5%)
HBV Hepatitis	29	447	476		93.9% (91.4%−95.7%)
Non-HBV Hepatitis	13	156	169		92.3% (87.3%−95.4%)
ND	6	122	128		95.3% (90.2%−97.8%)

*HBV* hepatitis B virus, *HCV* hepatitis C virus, *ND* patients with no detectable liver abnormalities, *CI* confidence interval, *BCLC* Barcelona Clinic Liver Cancer

### Independent test of the GAMAD model

To further evaluate the effectiveness of GAMAD, an independent test cohort was used that represented a full spectrum of patients in different clinical settings. Similar to the training and validation set, the GAMAD model demonstrated a sensitivity of 86.5% in HCC patients at stage 0-A. As expected, its sensitivity in HCC at stage B-C reached up to 91.7% (Table 1), with an AUC of 0.952 (95% CI, 0.931–0.973) in the test group (Fig. 4c). These results indicated that the superior performance of GAMAD in early-stage HCC detection also applied to advanced-stage HCC.

### Assessment of the GAMAD model

To further confirm the diagnostic capability of the GAMAD model for HCC, the widely recognized GALAD model was utilized as a benchmark for performance comparison. The sensitivity, specificity, and AUC of GALAD in the training, validation, and test sets were 80.5%, 83.9%, and 0.899 (95% CI, 0.871–0.928); 81.1%, 80.2%, and 0.906 (95% CI, 0.879–0.934); and 86.7%, 84.5%, and 0.926 (95% CI, 0.899–0.952), respectively (Table S8). All values were inferior to GAMAD (Fig. 4a-c and Table 1). The performance of GAMAD in both GALAD-positive and GALAD-negative samples was then assessed. GAMAD reduced 114 false positives among the 208 control individuals misclassified by GALAD. Furthermore, GAMAD maintained a detection rate of 40.2% in HCC cases that GALAD missed (Table S9).

Additionally, individual markers including AFP, DCP, AFP-L3, HepaAiQ, GALAD, and GAMAD were compared in terms of performance in the entire group, the cirrhosis subgroup, and the HBV subgroup. We found that GAMAD exhibited the highest performance, with sensitivities of 85.3%, 89.3%, and 86.1%, and AUCs of 0.945 (95% CI, 0.933–0.957, reference), 0.941 (95% CI, 0.924–0.957, reference), and 0.954 (95% CI, 0.941–0.966, reference), respectively. GALAD demonstrated the second-highest performance, with sensitivities of 82.8%, 86.1%, and 82.4%, and AUCs of 0.911 (95% CI, 0.895–0.927, *P* < 0.001), 0.902 (95% CI, 0.879–0.924, *P* < 0.001), and 0.914 (95% CI, 0.897–0.931, *P* < 0.001), respectively (Fig. 4d-f and Table S10-11). In the non-HBV subgroup, GAMAD demonstrated comparable sensitivity to GALAD (82.3% vs. 84.4%), but a significantly higher AUC (0.917, 95% CI: 0.882–0.951) than GALAD (0.897, 95% CI: 0.856–0.938, *P* < 0.001) (Fig. 4g and Table S12). GAMAD also demonstrated superior performance in distinguishing between early-stage HCC and stage 0 HCC within the subgroups of cirrhosis and HBV (Fig. S3). Subgroup analysis also showed comparable GAMAD performance between HBV patients receiving antiviral therapy (AUC 0.945, 95% CI 0.925–0.965) and those without (AUC 0.958, 95% CI 0.942–0.975), indicating that antiviral treatment status does not significantly impact the model’s diagnostic capability (Fig. S4, *P* = 0.311). Compared with tumor serum markers, while greatly improving the sensitivity, the specificity of GAMAD is only slightly behind. Furthermore, by fixing the specificity at 90% for sensitivity comparison, the GAMAD model was superior to individual markers and GALAD in the entire group, the cirrhosis subgroup, and the HBV subgroup (Table S13-15).

To assess the model improvement over GALAD, we evaluated GAMAD performance in the entire cohort using multiple metrics. At the established clinical high-risk cutoff, GAMAD correctly reclassified 7% more individuals into more appropriate risk categories than GALAD (categorical net reclassification index (NRI): 0.07, 95 CI: 0.031–0.109, *P* < 0.001; Table S16). Furthermore, GAMAD showed a superior risk stratification across the entire risk spectrum (continuous NRI: 0.959, 95 CI: 0.864–1.054, *P* < 0.001) and a gain of 8.7% in the average discrimination slope (integrated discrimination improvement (IDI): 0.087, 95 CI: 0.063–0.112, *P* < 0.001). The decision curve further demonstrated that GAMAD delivers an 18.9% improvement in average net benefit across most clinically relevant decision thresholds over GALAD (Fig. S5a and Table S16). Finally, GAMAD showed great agreement between predicted and observed probabilities (Fig. S5b-c). These results collectively confirm the superior performance of the GAMAD model over the GALAD.

**Fig. 5:**
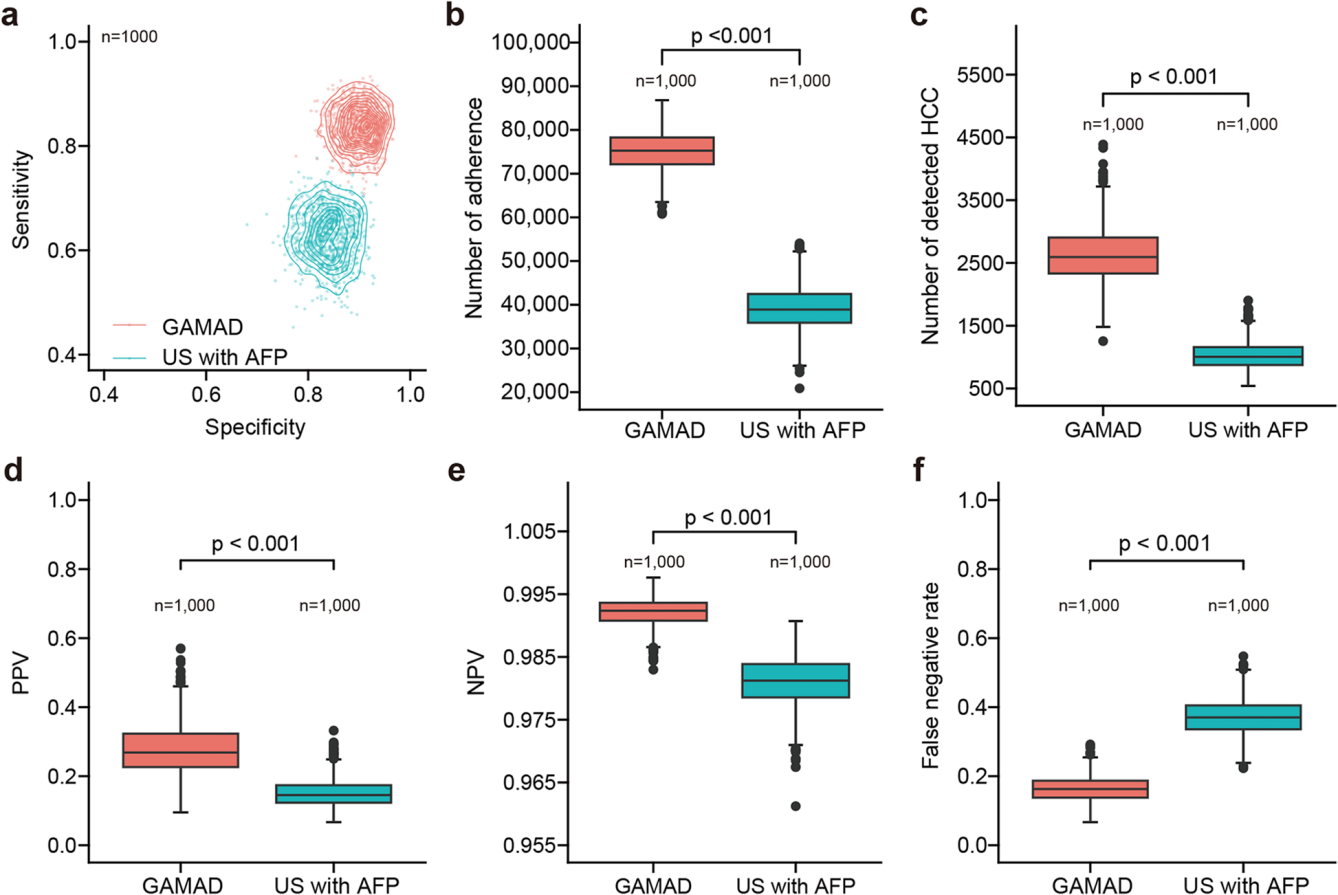
Modeling the implementation of GAMAD in early HCC screening. **a** The uncertainty of sensitivity and specificity of ultrasound (US) in combination with AFP (US with AFP) and the GAMAD model for screening in a theoretical population of 100,000 high-risk individuals. Predictive distributions for the number of adherence (**b**), detected HCC (**c**), positive predictive values (**d**), negative predictive values (**e**), and false negative rate (**f**), in a high-risk theoretical population. Mann–Whitney U-test, bars represent 95% CI. AFP: alpha-fetoprotein; HCC: hepatocellular carcinoma

Monte-Carlo simulations further demonstrated that the US combined with AFP identified an average of 1,030 individuals (95% CI, 657–1,519) with early HCC. Conversely, the GAMAD model enabled the detection of approximately 2,630 early HCC cases on average (95% CI, 1,879–3,563) (Fig. 5a-c and Table S17). Moreover, the positive predictive value (PPV) was expected to increase from 15.1% (95% CI, 9.2%–24.4%) to 28.0% (95% CI, 17.2%–44.3%), while the false predictive value decreased from 37.2% (95% CI, 27.8%–47.1%) to 16.3% (95% CI, 9.5%–24%). Additionally, the negative predictive value increased from 98.1% (95% CI, 97.3%−98.8%) to 99.2% (95% CI, 98.7%−99.6%, Fig. 5d-f and Table S17). Overall, GAMAD consistently outperformed GALAD and individual biomarkers across training, validation, and test sets, as well as in subgroup analyses. Monte-Carlo simulations also confirmed its superior population-level impact.

## Discussion

In this study, we prospectively recruited a large cohort of at-risk Chinese individuals for HCC developed a diagnostic model for early HCC called GAMAD. The novelty of GAMAD lies in its systematic integration of complementary biomarkers that reflect distinct biological aspects of HCC development. While each component has been studied individually, their optimal combination in GAMAD represents a significant advance in early HCC detection. A key consideration in developing GAMAD was ensuring its feasibility for widespread implementation, particularly in developing countries with high HCC burden. Rather than utilizing expensive genome-wide methylation analysis, our approach employs targeted qPCR methodology to analyze specific methylation sites.

Past research reveals differential methylation patterns in HCC tumor tissues compared with adjacent normal tissues and conditions such as cirrhosis or dysplastic nodules [17, 18]. Furthermore, numerous studies have demonstrated the effectiveness of ctDNA-based methylation markers in HCC diagnosis, achieving sensitivity rates ranging from 65 to 90%, and specificity rates from 81 to 94%, surpassing serum tumor markers [19–23]. In line with these observations, HepaAiQ, the ctDNA multi-gene methylation test used in our study, outperformed AFP, DCP, and AFP-L3 in HCC detection, especially in the early stage (BCLC 0-A). The sensitivity and AUC of HepaAiQ were the highest compared with AFP, DCP, and AFP-L3, while HepaAiQ had a specificity of 88.1%, lower than serum markers. This sacrifice in accuracy is normally observed in early cancer detection practice. In addition, while HepaAiQ provides information independent of AFP, L3, and DCP in multivariable analyses, the lower sensitivity observed in AFP-negative or DCP-negative HCC cases indicates that methylation status may still share some biological or clinical associations with conventional tumor markers.

We further established a model combining HepaAiQ, serum tumor markers, and clinical characteristics. Compared with the well-established model GALAD, GAMAD incorporates the ctDNA methylation assay HepaAiQ and excludes the serum AFP-L3 marker, which has been demonstrated in several studies to be neither an accurate nor specified marker [16, 24, 25]. This change improved model performance, with the sensitivity rising from 82.8% to 85.3%, the specificity rising from 82.9% to 90.4%, and the AUC rising from 0.911 to 0.945. Importantly, GALAD performed poorly in HBV populations [26], whereas GAMAD was developed using cohorts enriched for early-stage HCC, cirrhosis, hepatitis B, and non-HBV, making it more suitable for risk assessment in Chinese populations where HBV is a predominant etiological factor.

The implications of these findings are significant for clinical practice. GAMAD could be integrated into existing detection algorithms in several ways. First, in standard practice where US is the primary tool, GAMAD could be used as an adjunct diagnostic test for inconclusive or ambiguous US results. Second, in populations where US sensitivity is limited—such as patients with obesity or advanced cirrhosis—GAMAD could complement US by providing a molecular readout that is independent of operator skill or imaging quality. Third, in resource-limited settings where access to high-quality US is constrained, GAMAD has potential to act as a primary surveillance tool. Taken together, GAMAD can help to optimize detection strategies and ultimately improve outcomes in at-risk populations by serving as an adjunct diagnostic tool to US or as a replacement for US in resource-limited settings.

This study also has several limitations. First, the model was developed using a Chinese population; therefore, its performance and clinical applicability in other populations require further validation. The methylation signatures and other biomarkers included in GAMAD may perform differently in HCV-related or NAFLD-related HCC, as these conditions may involve distinct pathogenic mechanisms. Therefore, future international validation studies will help determine whether GAMAD can be directly applied to other populations or requires optimization of cut-off values and marker combinations. Second, although HepaAiQ was designed to target HCC-related methylation sites, we recognize that its score could potentially be influenced by occult or concurrent non-HCC malignancies. While patients with a known history of cancer were excluded, the possibility of subclinical or undiagnosed non-HCC malignancies cannot be entirely ruled out. Finally, while our current case–control study demonstrates GAMAD’s diagnostic accuracy, prospective longitudinal validation is needed to establish its performance in real-world screening scenarios.

In conclusion, GAMAD presents a promising diagnostic model that integrates gender, age, ctDNA methylation, and serum biomarkers to enhance the early detection of HCC. The model demonstrated performance superior to traditional markers and established models within a Chinese population. The incorporation of ctDNA methylation assays, specifically HepaAiQ, elevates diagnostic accuracy, particularly for early-stage HCC. While the high sensitivity and non-invasive nature of GAMAD suggest significant potential for population-wide screening, large-scale prospective validation and international multi-center studies are essential to confirm its generalizability and robustness prior to routine clinical implementation.

## Methods

### Patients and study design

This is a multi-center prospective observational trial (NCT05626985); the research protocol was previously published [27]. Individuals from both outpatient and inpatient settings were recruited, including HCC patients, patients with hepatitis or cirrhosis, and patients with no detectable liver abnormalities (ND). All HCC cases were newly diagnosed, treatment-naïve patients. HCC stage was performed according to the Barcelona Clinic Liver Cancer (BCLC) criteria by a multidisciplinary team including hepatologists, radiologists, and liver surgeons (Supplementary methods). HBV patients were managed according to current guidelines, of which 646 received antiviral treatment and 465 did not receive treatment. Importantly, patients enrolled with hepatitis or cirrhosis were required not to develop HCC within six months of enrollment. The trial received approval from the ethical committees of the hospitals, including the Eastern Hepatobiliary Surgery Hospital, Naval Medical University (EHBHKY2023-H0003-P001), the First Hospital of Jilin University (22K073-001), and Tianjin Third Central Hospital (IRB2023-007–01).

To ensure analytical independence and minimize potential bias from commercial affiliations, the following measures were strictly implemented: (1) Sample collection was performed independently by clinicians and staff from three centers, while sample processing and testing were conducted independently by personnel unaware of the clinical information; (2) the analysts for the validation and independent test sets remained blinded to the clinical information prior to unblinding; and (3) all authors had full access to the study data, and reviewed and approved the final manuscript.

### Blood test

About 10 mL peripheral blood samples were individually collected from various centers before the initial diagnosis to test HepaAiQ, AFP, DCP, and AFP-L3, which have been described in detail in the research protocol [27]. Briefly, circulating tumor DNA methylation was measured using the HepaAiQ assay kit (EU CE certification #NL-CA002-2022–68891, Supplementary methods). AFP, DCP, and AFP-L3 were all measured in the same serum sample. The measurements were undertaken using a commercial kit (Beijing Hotgen BioTech Co., Ltd.) through chemiluminescent magnetic particle immunoassay methodology. Throughout the measurement process, diagnostic and clinical information, including the disease status of each sample, was masked until the results were ready for statistical analysis. All sites followed the same standard operating procedures for blood sample collection, processing, and DNA extraction. Furthermore, methylation testing was performed using identical instrument models and assay kits, with all laboratory personnel trained under the same protocol prior to study initiation.

### Construction and validation of the GAMAD model

To assess associations with HCC, odds ratios were derived using logistic regression in univariate and multivariate analyses. Variables such as HepaAiQ, gender, age, AFP, AFP-L3, and DCP, were considered for inclusion in the multivariable models. Receiver operating characteristic (ROC) curves were used to evaluate the predictive accuracy of the model. Subsequently, we utilized Gender, Age, DNA Methylation (HepaAiQ score), AFP, and DCP as variables to develop a nomogram-based calculator in the training cohort, designated as the GAMAD model. The logistic regression formula for the GAMAD model is given by: GAMAD logit(p) score = −6.5871 + 1.3776 × [Gender (0 for female, 1 for male)] + 0.0719 × [Age] + 0.3647 × ln [AFP] + 0.7860 × ln [DCP] + 1.8365 × [HepaAiQ score]. The cutoff of the logit(p) score, according to the Youden index, was −1.057373.

The incremental diagnostic value of GAMAD over GALAD was assessed using IDI and NRI (calculated with R's PredictABEL package). Clinical utility was evaluated via calibration curves and decision curve analysis, with calibration analyses conducted using the rms package in R.

### Monte Carlo simulation

Monte-Carlo simulations were used to compare the GAMAD approach with US combined with AFP in a theoretical population. The simulation parameters for ultrasound combined with AFP were derived from a comprehensive meta-analysis by Tzartzeva et al. [28], which reported a pooled sensitivity of 63% (95% CI, 48%−75%) and specificity of 84% (95% CI, 77%−89%) for early-stage HCC detection. For GAMAD, we used performance metrics from our validation cohort (sensitivity 80.5%, specificity 90.4%) to ensure conservative estimates based on actual performance data rather than theoretical values. The simulation incorporated published HCC incidence rates in Chinese cirrhotic patients (3–5% annually) and reported surveillance adherence rates (65–85%) from real-world studies in China. Multinomial probabilities were modeled for the prevalence of viral hepatitis, cirrhosis, viral hepatitis + HCC, cirrhosis + HCC, and viral hepatitis + cirrhosis + HCC from a Dirichlet distribution with parameters 890, 68, 18, 6, and 18, respectively [29, 30]. Monte Carlo simulation was repeated 1,000 times to evaluate the performance parameters.

### Statistical analysis

Continuous variables were compared using the Mann–Whitney U test, while categorical variables were compared using the Chi-square test. The DeLong's test was used to compare two correlated ROC curves. Statistical analyses were performed using SPSS software version 22.0 and R version 4.13. All *p*-values were two-sided, with values less than 0.05 considered statistically significant.

## Supplementary Information

Supplementary Material 1.

## Data Availability

The data that support the findings of this study are available in the supplementary material of this article. Further inquiries can be directed to the corresponding author.
