# Supplementary material for: Development and validation of a high-confidence diagnostic model integrating ctDNA methylation and serum biomarkers for early-stage hepatocellular carcinoma detection
Source: Mol Biomed. 2026 Mar 11;7:26. doi: 10.1186/s43556-026-00426-3 (PMC12976210; doi:10.1186/s43556-026-00426-3)
Supplement: Supplementary file 1 — Supplementary Material 1. [file 43556_2026_426_MOESM1_ESM.docx]

**Development and validation of a high-confidence diagnostic model integrating ctDNA methylation and serum biomarkers for early-stage hepatocellular carcinoma detection**

Han Wu^1,†^, Mingda Wang^1,†^, Zhiyi Wan^2,†^, Lanqing Yao^1,†^, Shuang Zhou^2^, Hui Wang^2^, Guoyue Lv^3^, Nanya Wang^4^, Fengmei Wang^5^, Jiahao Xu^1^, Xinfei Xu^1^, Chao Li^1^, Yongkang Diao^1^, Timohty M. Pawlik^6^, Rui Liu^2,^*, Feng Shen^1,^*, and Tian Yang^1,^*

**Table of contents**

[**Supplementary methods** 2](#_Toc203728928)

[**Supplementary figures** 4](#_Toc203728929)

[**Supplementary tables** 10](#_Toc203728930)

[**References** 38](#_Toc203728931)

# **Supplementary methods**

**Patient Stage**

HCC stage was determined according to the Barcelona Clinic Liver Cancer (BCLC) criteria. Specifically, stage assessment included: 1) Dynamic contrast-enhanced CT or MRI with standardized reporting, 2) Chest CT to evaluate extrahepatic spread, 3) Laboratory tests including AFP and other relevant markers, 4) Performance status evaluation, 5) Child-Pugh classification of liver function, and 6) Histopathological confirmation when available. Two independent reviewers assessed all cases, with disagreements resolved by consensus discussion.

**HepaAiQ assay**

Based on previous research [1], we performed lasso regression analysis on 20 candidate methylation markers to identify the most relevant features for diagnostic modelling, including 7 methylation markers (*IKZF1*, *Septin9*, *Septin9_region2*, *B4GALNT1*, *BEST4*, *BEND4,* and *GRASP*). The Ct values for each marker were normalised using predefined minimum (Ctmin = 15) and maximum (Ctmax = 40) Ct values as reference points. The normalisation formula used was Normalised Ct = (Cttest-Ctmin)/(Ctmax-Ctmin). The logistic regression formula based on the normalised Ct values is expressed as follows: logit(𝑃) = 4.85-0.97×*IKZF1*-1.46×*Septin9*-0.94×*Septin9_region2*-0.73×*B4GALNT1*-0.9×*BEST4*-0.96×*BEND4*-0.35×*GRASP*. The cut-off value for logit(P) is 0.26. A result above this threshold is considered positive.

**HepaAiQ assay reproducibility analysis**

Intra-assay (two replicates) and inter-assay (two replicates across two runs over 20 consecutive days by two operators on three instruments) precision of the detected methylation markers in the HepaAiQ assay were evaluated using a panel of three methylated DNA samples—consisting of 2% methylated DNA, 1% methylated DNA, and 0% methylated DNA—across three independent reagent lots. Intra- and inter-assay variations were calculated from the mean Ct values and expressed as coefficients of variation (CV).

# **Supplementary figures**


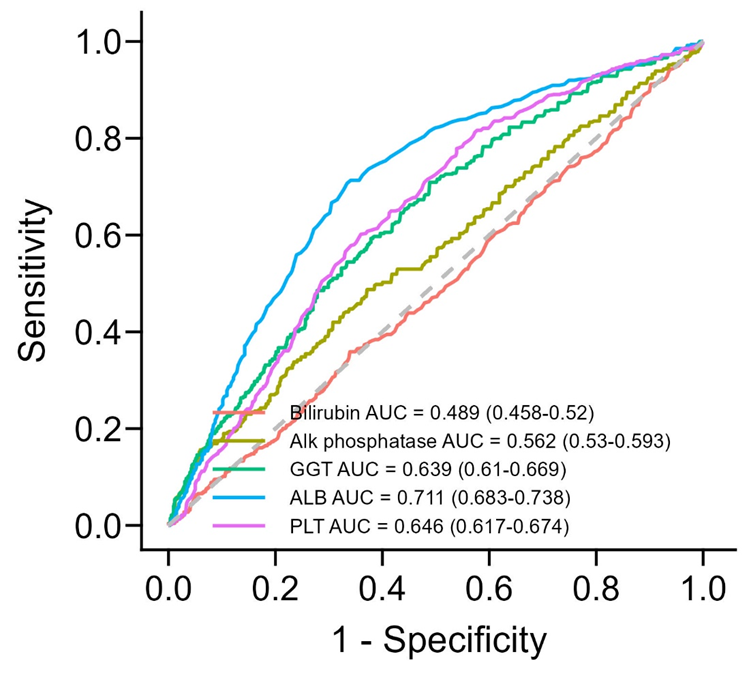


**Figure 1. Performance of** **biochemistry parameters in the entire cohort.** GGT, Gamma-glutamyl transferase; ALB, Albumin; PLT, Platelet. DeLong's test.


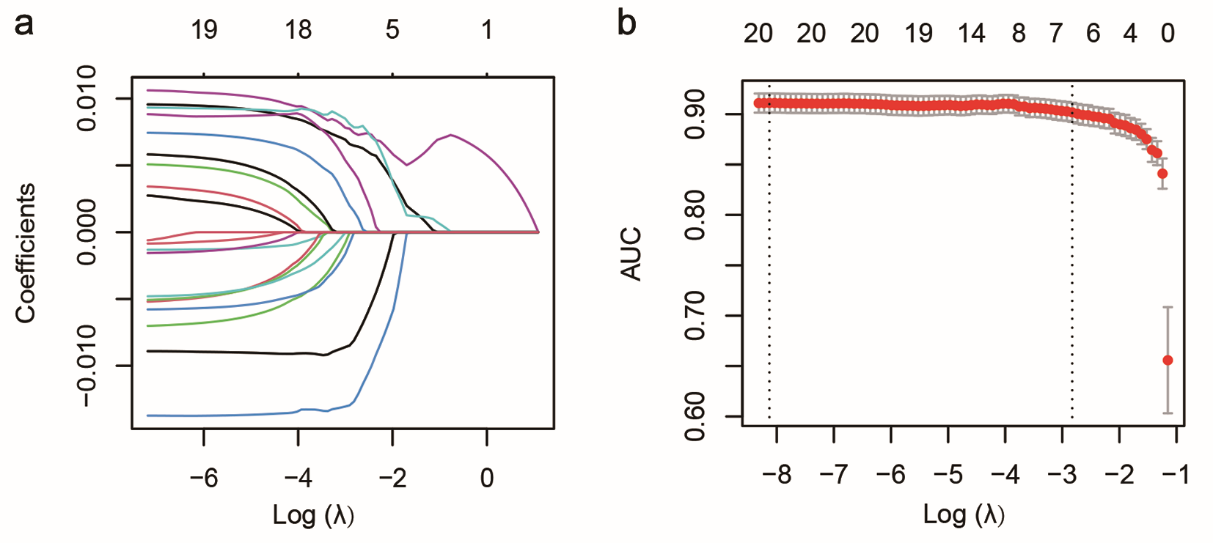


**Figure 2. Feature selection was conducted using the least absolute shrinkage and selection operator (LASSO) binary logistic regression model.** (a) The LASSO coefficient profiles for 20 methylation markers are shown. Each curve represents the trajectory of a marker's coefficient as the regularization penalty parameter (λ) changes, with the x-axis indicating log(λ). (b) A 10-fold cross-validation was performed to identify the optimal λ. The area under the receiver operating characteristic (AUC) curve was plotted against log(λ).


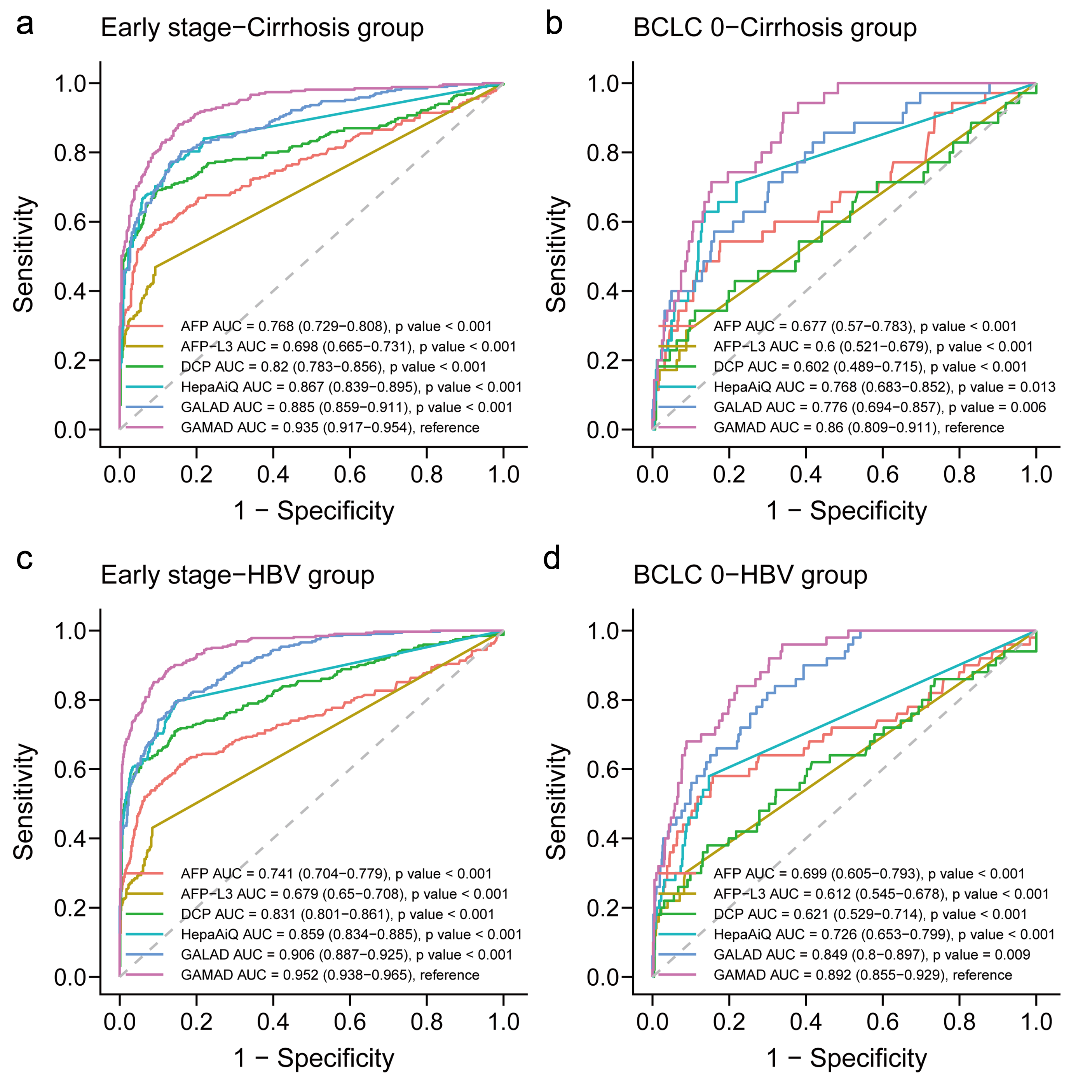


**Figure 3. Comparison of diagnostic performance of individual markers and models for early HCC and stage 0 HCC in cirrhosis (a, b) and HBV (c, d) subgroups.** DeLong's test.


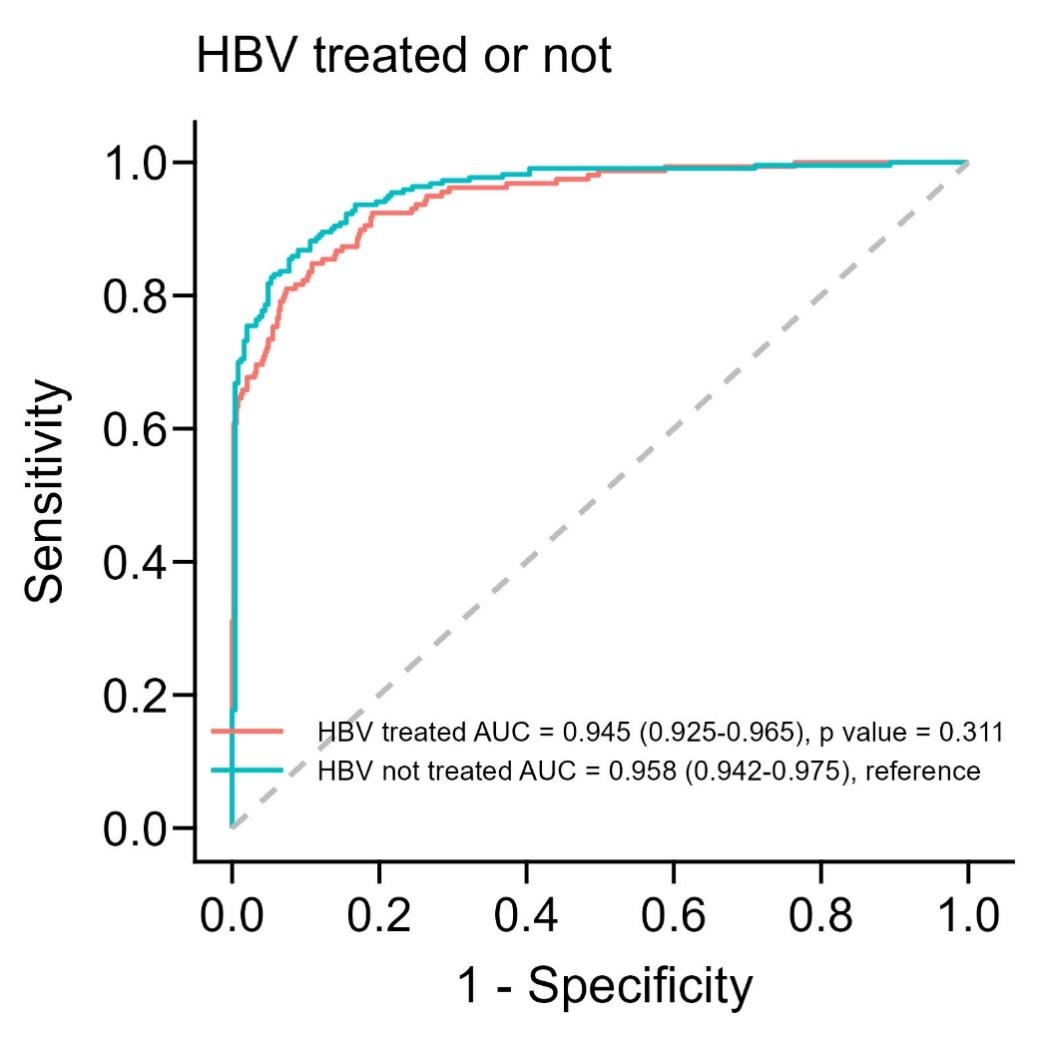


**Figure 4. Comparison of GAMAD model performance in treated and untreated HBV patients.** DeLong's test.


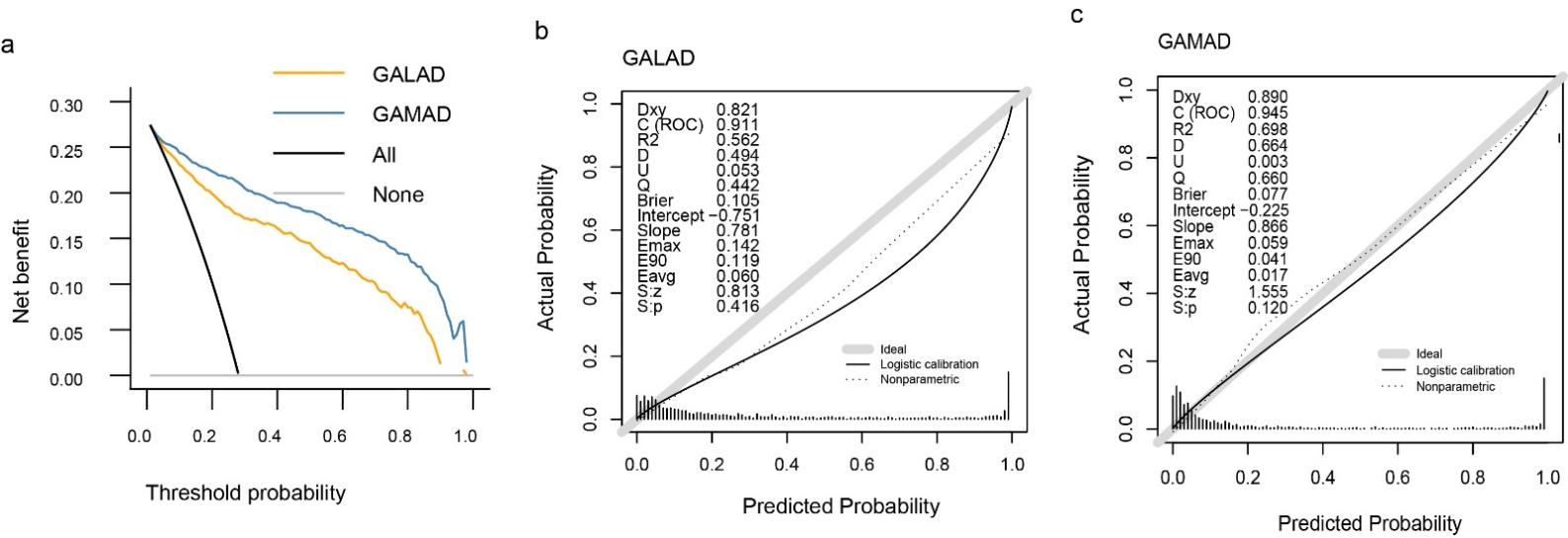


**Figure 5. Comparison of clinical utility calibration and between GAMAD and GALAD models in the overall cohort.** (a) Decision curve analysis (DCA) comparing the clinical net benefit of the GAMAD and GALAD models across a range of threshold probabilities. The orange and blue curves show the net benefit for each model. The grey horizontal line represents the "treat none" strategy (net benefit = 0), and the black diagonal line represents the "treat all" strategy. The model with the highest net benefit at a clinically relevant threshold range demonstrates superior clinical utility. Calibration curves of the GALAD (b) and GAMAD (c) models. Dashed curve: non-parametric estimate of the calibration relationship between actual and predicted probability, grey line: ideal relationship (intercept of zero and slope of one). Dxy: Somer's rank correlation, C(ROC): AUC for discrimination, R2: Nagelkerke-Cox-Snell-Maddala-Magee R-squared index, D: discrimination index, U: unreliability index, Q: quality index, Brier: Brier score (average squared difference in predicted and actual probabilities), Emax/E90/Eavg: Maximum/90 th quantile, average absolute difference in predicted and smoothed calibrated probabilities, S:z/S:p the z and two sided p-value of the Spiegelhalter test for calibration accuracy. Graphs generated by the "val.prob" function in the RMS R-package. All analyses were performed using the entire cohort (n = 1,692).

# **Supplementary tables**

**Table 1. Clinicopathological characteristics of the HCC group and the control group.**

| **Variable** | **All (N = 1,692)** | |
| --- | --- | --- |
|  | **Cancer (N = 476)** | **Control (N = 1,216)** |
| Age |  |  |
| Median (Min, Max) | 58.0 (15.0, 81.0) | 49.0 (18.0, 85.0) |
| Gender |  |  |
| Female | 86 (18.1%) | 463 (38.1%) |
| Male | 390 (81.9%) | 753 (61.9%) |
| Cirrhosis |  |  |
| No | 138 (29.0%) | 773 (63.6%) |
| Yes | 338 (71.0%) | 443 (36.4%) |
| Etiology of liver disease |  |  |
| HBV | 380 (79.8%) | 790 (65.0%) |
| Non-HBV | 96 (20.2%) | 298 (24.5%) |
| Healthy | 0 (0%) | 128 (10.5%) |
| Tumor size (cm) |  |  |
| <5 | 292 (61.3%) |  |
| ≥5 | 184 (38.7%) |  |
| Tumor number |  |  |
| Single | 376 (79.0%) |  |
| Multiple | 100 (21.0%) |  |
| Gross vascular invasion |  |  |
| No | 431 (90.5%) |  |
| Yes | 45 (9.5%) |  |
| Distant metastasis |  |  |
| No | 461 (96.8%) |  |
| Yes | 15 (3.2%) |  |
| BCLC stage |  |  |
| 0 | 65 (13.7%) |  |
| A | 327 (68.7%) |  |
| B/C | 84 (17.6%) |  |
| Control type |  |  |
| Cirrhosis |  | 443 (36.4%) |
| HBV Hepatitis |  | 476 (39.1%) |
| Non-HBV Hepatitis |  | 169 (13.9%) |
| ND |  | 128 (10.5%) |

HBV: hepatitis B virus; BCLC: Barcelona Clinic Liver Cancer; ND; patients with no detectable liver abnormalities.

**Table 2. Baseline information of training and validation cohorts.**

| Variable | Cancer (N=318) | |  | Control (N=809) | |  |
| --- | --- | --- | --- | --- | --- | --- |
|  | Training (N=159) | Validation (N=159) | P value | Training (N=404) | Validation (N=405) | P value |
| Age |  |  | 0.866 |  |  | 0.301 |
| Median (Min, Max) | 58.0 (15.0, 76.0) | 58.0 (34.0, 77.0) |  | 49.0 (18.0, 85.0) | 48.0 (18.0, 74.0) |  |
| Gender |  |  | 0.669 |  |  | 0.104 |
| Female | 29 (18.2%) | 32 (20.1%) |  | 162 (40.1%) | 140 (34.6%) |  |
| Male | 130 (81.8%) | 127 (79.9%) |  | 242 (59.9%) | 265 (65.4%) |  |
| Cirrhosis |  |  | 0.149 |  |  | 0.963 |
| No | 45 (28.3%) | 57 (35.8%) |  | 257 (63.6%) | 257 (63.5%) |  |
| Yes | 114 (71.7%) | 102 (64.2%) |  | 147 (36.4%) | 148 (36.5%) |  |
| Etiology of liver disease |  |  | 0.471 |  |  | 0.383 |
| HBV | 127 (79.9%) | 132 (83.0%) |  | 254 (62.9%) | 272 (67.2%) |  |
| Non-HBV | 32 (20.1%) | 27 (17.0%) |  | 43 (10.6%) | 42 (10.4%) |  |
| Healthy |  |  |  | 107 (26.5%) | 91 (22.5%) |  |
| Tumor size (cm) |  |  | 0.906 |  |  |  |
| <5 | 105 (66.0%) | 104 (65.4%) |  |  |  |  |
| ≥5 | 54 (34.0%) | 55 (34.6%) |  |  |  |  |
| Tumor. Number |  |  | 0.032 |  |  |  |
| Single | 139 (87.4%) | 150 (94.3%) |  |  |  |  |
| Multiple | 20 (12.6%) | 9 (5.7%) |  |  |  |  |
| Gross vascular invasion |  |  | 1 |  |  |  |
| No | 158 (99.4%) | 159 (100%) |  |  |  |  |
| Yes | 1 (0.6%) | 0 (0%) |  |  |  |  |
| Distant metastasis |  |  | 1 |  |  |  |
| No | 159 (100%) | 159 (100%) |  |  |  |  |
| Yes | 0 (0%) | 0 (0%) |  |  |  |  |
| BCLC stage |  |  | 0.88 |  |  |  |
| 0 | 26 (16.4%) | 27 (17.0%) |  |  |  |  |
| A | 133 (83.6%) | 132 (83.0%) |  |  |  |  |
| B/C | 0 (0%) | 0 (0%) |  |  |  |  |
| Control type |  |  |  |  |  | 0.999 |
| Cirrhosis |  |  |  | 147 (36.4%) | 148 (36.5%) |  |
| HBV Hepatitis |  |  |  | 158 (39.1%) | 159 (39.3%) |  |
| Non-HBV Hepatitis |  |  |  | 56 (13.9%) | 56 (13.8%) |  |
| ND |  |  |  | 43 (10.6%) | 42 (10.4%) |  |

Abbreviation: HBV: hepatitis B virus; HCV: hepatitis C virus; BCLC: Barcelona Clinic Liver Cancer; ND; patients with no detectable liver abnormalities. Continuous variables: Mann-Whitney U-test; categorical variables: Chi-square test.

**Table 3. Clinicopathological characteristics of the training, validation, and test cohorts.**

| Variable | Training (N=563) | | | Validation (N=564) | | | Test (N= 565) | | |
| --- | --- | --- | --- | --- | --- | --- | --- | --- | --- |
|  | Cancer (N=159) | Control (N=404) | P value | Cancer (N=159) | Control (N=405) | P value | Cancer (N=158) | Control (N=407) | P value |
| Age |  |  | <0.001 |  |  | <0.001 |  |  | <0.001 |
| Median (Min, Max) | 58.0 (15.0, 76.0) | 49.0 (18.0, 85.0) |  | 58.0 (34.0, 77.0) | 48.0 (18.0, 74.0) |  | 59.0 (29.0, 81.0) | 50.0 (18.0, 78.0) |  |
| Gender |  |  | <0.001 |  |  | <0.001 |  |  | <0.001 |
| Female | 29 (18.2%) | 162 (40.1%) |  | 32 (20.1%) | 140 (34.6%) |  | 25 (15.8%) | 161 (39.6%) |  |
| Male | 130 (81.8%) | 242 (59.9%) |  | 127 (79.9%) | 265 (65.4%) |  | 133 (84.2%) | 246 (60.4%) |  |
| AFP (ng/mL) |  |  | <0.001 |  |  | <0.001 |  |  | <0.001 |
| Median (Min, Max) | 14.9 (1.23, 1200) | 3.67 (1.11, 485) |  | 11.7 (1.10, 1200) | 3.49 (1.00, 1150) |  | 52.8 (0.600, 1200) | 3.46 (0.730, 329) |  |
| AFP-L3 (%) |  |  | <0.001 |  |  | <0.001 |  |  | <0.001 |
| Median (Min, Max) | 5.00 (5.00, 50.0) | 5.00 (5.00, 25.7) |  | 5.00 (5.00, 50.0) | 5.00 (5.00, 23.4) |  | 9.90 (5.00, 50.0) | 5.00 (5.00, 17.2) |  |
| DCP (ng/mL) |  |  | <0.001 |  |  | <0.001 |  |  | <0.001 |
| Median (Min, Max) | 38.4 (1.30, 20000) | 5.00 (2.03, 70.0) |  | 32.4 (1.30, 20000) | 4.81 (2.03, 9560) |  | 165 (1.30, 20000) | 4.56 (2.01, 779) |  |
| ln (AFP) |  |  | <0.001 |  |  | <0.001 |  |  | <0.001 |
| Median (Min, Max) | 2.70 (0.207, 7.09) | 1.30 (0.104, 6.18) |  | 2.46 (0.0953, 7.09) | 1.25 (0, 7.05) |  | 3.97 (-0.511, 7.09) | 1.24 (-0.315, 5.80) |  |
| ln (DCP) |  |  | <0.001 |  |  | <0.001 |  |  | <0.001 |
| Median (Min, Max) | 3.65 (0.262, 9.90) | 1.61 (0.708, 4.25) |  | 3.48 (0.262, 9.90) | 1.57 (0.708, 9.17) |  | 5.10 (0.262, 9.90) | 1.52 (0.698, 6.66) |  |
| AFP range (ng/mL) |  |  | <0.001 |  |  | <0.001 |  |  | <0.001 |
| <20 | 85 (53.5%) | 383 (94.8%) |  | 89 (56.0%) | 389 (96.0%) |  | 61 (38.6%) | 393 (96.6%) |  |
| ≥20 | 74 (46.5%) | 21 (5.2%) |  | 70 (44.0%) | 16 (4.0%) |  | 97 (61.4%) | 14 (3.4%) |  |
| AFP_L3_range (%) |  |  | <0.001 |  |  | <0.001 |  |  | <0.001 |
| <10 | 104 (65.4%) | 384 (95.0%) |  | 104 (65.4%) | 380 (93.8%) |  | 79 (50.0%) | 382 (93.9%) |  |
| ≥10 | 55 (34.6%) | 20 (5.0%) |  | 55 (34.6%) | 25 (6.2%) |  | 79 (50.0%) | 25 (6.1%) |  |
| DCP range (ng/mL) |  |  | <0.001 |  |  | <0.001 |  |  | <0.001 |
| <40 | 81 (50.9%) | 399 (98.8%) |  | 83 (52.2%) | 395 (97.5%) |  | 58 (36.7%) | 393 (96.6%) |  |
| ≥40 | 78 (49.1%) | 5 (1.2%) |  | 76 (47.8%) | 10 (2.5%) |  | 100 (63.3%) | 14 (3.4%) |  |
| HepaAiQ score |  |  | <0.001 |  |  | <0.001 |  |  | <0.001 |
| Median (Min, Max) | -0.0260 (-1.46, 3.91) | -1.46 (-1.46, 0.628) |  | -0.446 (-1.46, 3.65) | -1.46 (-1.46, 2.06) |  | 0.737 (-1.46, 4.03) | -1.46 (-1.46, 1.48) |  |
| HepaAiQ call |  |  | <0.001 |  |  | <0.001 |  |  | <0.001 |
| Negative | 37 (23.3%) | 358 (88.6%) |  | 53 (33.3%) | 362 (89.4%) |  | 31 (19.6%) | 351 (86.2%) |  |
| Positive | 122 (76.7%) | 46 (11.4%) |  | 106 (66.7%) | 43 (10.6%) |  | 127 (80.4%) | 56 (13.8%) |  |
| Cirrhosis |  |  | <0.001 |  |  | <0.001 |  |  | <0.001 |
| No | 45 (28.3%) | 257 (63.6%) |  | 57 (35.8%) | 257 (63.5%) |  | 36 (22.8%) | 259 (63.6%) |  |
| Yes | 114 (71.7%) | 147 (36.4%) |  | 102 (64.2%) | 148 (36.5%) |  | 122 (77.2%) | 148 (36.4%) |  |
| Etiology of liver disease |  |  | <0.001 |  |  | <0.001 |  |  | <0.001 |
| HBV | 127 (79.9%) | 254 (62.9%) |  | 132 (83.0%) | 272 (67.2%) |  | 121 (76.6%) | 264 (64.9%) |  |
| Non-HBV | 32 (20.1%) | 107 (26.5%) |  | 27 (17.0%) | 91 (22.5%) |  | 37 (23.4%) | 100 (24.6%) |  |
| Healthy | 0 (0%) | 43 (10.6%) |  | 0 (0%) | 42 (10.4%) |  | 0 (0%) | 43 (10.6%) |  |
| Tumor size (cm) |  |  |  |  |  |  |  |  |  |
| <5 | 105 (66.0%) | 0 (0%) |  | 104 (65.4%) | 0 (0%) |  | 83 (52.5%) | 0 (0%) |  |
| ≥5 | 54 (34.0%) | 0 (0%) |  | 55 (34.6%) | 0 (0%) |  | 75 (47.5%) | 0 (0%) |  |
| Tumor. Number |  |  |  |  |  |  |  |  |  |
| Single | 139 (87.4%) |  |  | 150 (94.3%) |  |  | 87 (55.1%) |  |  |
| Multiple | 20 (12.6%) |  |  | 9 (5.7%) |  |  | 71 (44.9%) |  |  |
| Gross vascular invasion |  |  |  |  |  |  |  |  |  |
| No | 158 (99.4%) |  |  | 159 (100%) |  |  | 114 (72.2%) |  |  |
| Yes | 1 (0.6%) |  |  | 0 (0%) |  |  | 44 (27.8%) |  |  |
| Distant metastasis |  |  |  |  |  |  |  |  |  |
| No | 159 (100%) |  |  | 159 (100%) |  |  | 143 (90.5%) |  |  |
| Yes | 0 (0%) |  |  | 0 (0%) |  |  | 15 (9.5%) |  |  |
| BCLC stage |  |  |  |  |  |  |  |  |  |
| 0 | 26 (16.4%) |  |  | 27 (17.0%) |  |  | 12 (7.6%) |  |  |
| A | 133 (83.6%) |  |  | 132 (83.0%) |  |  | 62 (39.2%) |  |  |
| B/C | 0 (0%) |  |  | 0 (0%) |  |  | 84 (53.2%) |  |  |
| Control type |  |  |  |  |  |  |  |  |  |
| Cirrhosis |  | 147 (36.4%) |  |  | 148 (36.5%) |  |  | 148 (36.4%) |  |
| HBV Hepatitis |  | 158 (39.1%) |  |  | 159 (39.3%) |  |  | 159 (39.1%) |  |
| Non-HBV Hepatitis |  | 56 (13.9%) |  |  | 56 (13.8%) |  |  | 57 (14.0%) |  |
| ND |  | 43 (10.6%) |  |  | 42 (10.4%) |  |  | 43 (10.6%) |  |

Abbreviation: AFP: alpha-fetoprotein; AFP-L3: Lens culinaris agglutinin A-reactive fraction of alpha-fetoprotein; DCP: Des-γ-carboxy-prothrombin; HBV: hepatitis B virus; HCV: hepatitis C virus; BCLC: Barcelona Clinic Liver Cancer; ND; patients with no detectable liver abnormalities. Continuous variables: Mann-Whitney U-test; categorical variables: Chi-square test.

**Table 4. Intra- and Inter-assay reproducibility of the HepaAiQ assay.**

| Reagent lots | Markers | Methylation ratio of DNA | Intra-assay | | | Inter-assay | | |
| --- | --- | --- | --- | --- | --- | --- | --- | --- |
|  |  |  | Mean | SD | CV (%) | Mean | SD | CV (%) |
| LOT1 | *IKZF1* | *2%* | *19.1* | *0.004* | *0.023* | *19.1* | *0.387* | *2.024* |
|  |  | 1% | 20.0 | 0.013 | 0.065 | 20.0 | 0.473 | 2.360 |
|  |  | 0% | 40 | 0 | 0 | 40.0 | 0 | 0 |
|  | *Septin9* | 2% | 20.4 | 0.015 | 0.073 | 20.2 | 0.342 | 1.692 |
|  |  | 1% | 21.3 | 0.030 | 0.139 | 21.3 | 0.474 | 2.229 |
|  |  | 0% | 40 | 0 | 0 | 40.0 | 0 | 0 |
|  | *Septin9_region2* | 2% | 21.4 | 0.111 | 0.516 | 21.4 | 0.290 | 1.352 |
|  |  | 1% | 22.4 | 0.022 | 0.100 | 22.4 | 0.362 | 1.616 |
|  |  | 0% | 40 | 0 | 0 | 40.0 | 0 | 0 |
|  | *B4GALNT1* | 2% | 22.3 | 0.042 | 0.188 | 22.2 | 0.352 | 1.584 |
|  |  | 1% | 23.3 | 0.006 | 0.027 | 23.2 | 0.494 | 2.130 |
|  |  | 0% | 40 | 0 | 0 | 40.0 | 0 | 0 |
|  | *BEST4* | 2% | 22.7 | 0.030 | 0.132 | 23.0 | 0.441 | 1.912 |
|  |  | 1% | 23.8 | 0.009 | 0.039 | 24.1 | 0.485 | 2.011 |
|  |  | 0% | 40 | 0 | 0 | 40.0 | 0 | 0 |
|  | *BEND4* | 2% | 22.2 | 0.043 | 0.195 | 22.3 | 0.364 | 1.636 |
|  |  | 1% | 23.4 | 0.121 | 0.515 | 23.3 | 0.393 | 1.686 |
|  |  | 0% | 40 | 0 | 0 | 40.0 | 0 | 0 |
|  | *GRASP* | 2% | 22.0 | 0.011 | 0.048 | 22.2 | 0.402 | 1.815 |
|  |  | 1% | 23.2 | 0.053 | 0.227 | 23.1 | 0.367 | 1.590 |
|  |  | 0% | 40 | 0 | 0 | 40.0 | 0 | 0 |
| LOT2 | *IKZF1* | 2% | 19.4 | 0.072 | 0.370 | 19.3 | 0.318 | 1.653 |
|  |  | 1% | 20.2 | 0.032 | 0.160 | 20.1 | 0.341 | 1.698 |
|  |  | 0% | 40 | 0 | 0 | 40.0 | 0 | 0 |
|  | *Septin9* | 2% | 20.6 | 0.038 | 0.185 | 20.3 | 0.357 | 1.757 |
|  |  | 1% | 21.3 | 0.075 | 0.352 | 21.4 | 0.456 | 2.127 |
|  |  | 0% | 40 | 0 | 0 | 40.0 | 0 | 0 |
|  | *Septin9_region2* | 2% | 21.2 | 0.048 | 0.229 | 21.5 | 0.377 | 1.753 |
|  |  | 1% | 22.0 | 0.015 | 0.069 | 22.2 | 0.369 | 1.662 |
|  |  | 0% | 40 | 0 | 0 | 40.0 | 0 | 0 |
|  | *B4GALNT1* | 2% | 22.1 | 0.074 | 0.336 | 22.2 | 0.321 | 1.446 |
|  |  | 1% | 22.9 | 0.014 | 0.063 | 23.0 | 0.440 | 1.916 |
|  |  | 0% | 40 | 0 | 0 | 40.0 | 0 | 0 |
|  | *BEST4* | 2% | 23.0 | 0.024 | 0.106 | 23.1 | 0.601 | 2.607 |
|  |  | 1% | 23.7 | 0.128 | 0.540 | 24.1 | 0.535 | 2.224 |
|  |  | 0% | 40 | 0 | 0 | 40.0 | 0 | 0 |
|  | *BEND4* | 2% | 22.2 | 0.023 | 0.103 | 22.4 | 0.481 | 2.147 |
|  |  | 1% | 23.3 | 0.149 | 0.641 | 23.3 | 0.420 | 1.802 |
|  |  | 0% | 40 | 0 | 0 | 40.0 | 0.114 | 0.286 |
|  | *GRASP* | 2% | 22.1 | 0.017 | 0.077 | 22.2 | 0.285 | 1.287 |
|  |  | 1% | 23.1 | 0.169 | 0.732 | 23.1 | 0.346 | 1.495 |
|  |  | 0% | 40 | 0 | 0 | 40.0 | 0 | 0 |
| LOT3 | *IKZF1* | 2% | 19.4 | 0.072 | 0.370 | 19.3 | 0.318 | 1.653 |
|  |  | 1% | 20.2 | 0.032 | 0.160 | 20.1 | 0.341 | 1.698 |
|  |  | 0% | 40 | 0 | 0 | 40.0 | 0 | 0 |
|  | *Septin9* | 2% | 20.6 | 0.038 | 0.185 | 20.3 | 0.357 | 1.757 |
|  |  | 1% | 21.3 | 0.075 | 0.352 | 21.4 | 0.456 | 2.127 |
|  |  | 0% | 40 | 0 | 0 | 40.0 | 0 | 0 |
|  | *Septin9_region2* | 2% | 21.2 | 0.048 | 0.229 | 21.5 | 0.377 | 1.753 |
|  |  | 1% | 22.0 | 0.015 | 0.069 | 22.2 | 0.369 | 1.662 |
|  |  | 0% | 40 | 0 | 0 | 40.0 | 0 | 0 |
|  | *B4GALNT1* | 2% | 22.1 | 0.074 | 0.336 | 22.2 | 0.321 | 1.446 |
|  |  | 1% | 22.9 | 0.014 | 0.063 | 23.0 | 0.440 | 1.916 |
|  |  | 0% | 40 | 0 | 0 | 40.0 | 0 | 0 |
|  | *BEST4* | 2% | 23.0 | 0.024 | 0.106 | 23.1 | 0.601 | 2.607 |
|  |  | 1% | 23.7 | 0.128 | 0.540 | 24.1 | 0.535 | 2.224 |
|  |  | 0% | 40 | 0 | 0 | 40.0 | 0 | 0 |
|  | *BEND4* | 2% | 22.2 | 0.023 | 0.103 | 22.4 | 0.481 | 2.147 |
|  |  | 1% | 23.3 | 0.149 | 0.641 | 23.3 | 0.420 | 1.802 |
|  |  | 0% | 40 | 0 | 0 | 40.0 | 0.114 | 0.286 |
|  | *GRASP* | 2% | 22.1 | 0.017 | 0.077 | 22.2 | 0.285 | 1.287 |
|  |  | 1% | 23.1 | 0.169 | 0.732 | 23.1 | 0.346 | 1.495 |
|  |  | 0% | 40 | 0 | 0 | 40.0 | 0 | 0 |

Abbreviation: HBV: SD: Standard Deviation; CV: Coefficient of Variation.

**Table 5. Diagnostic performances of HepaAiQ, AFP, AFP-L3, and DCP for HCC detection.**

| **Sample type** | **Positive** | **Negative** | **Total** | **Sensitivity (95% CI)** | **Specificity (95% CI)** |
| --- | --- | --- | --- | --- | --- |
| **HepaAiQ (N/P)** |  |  |  |  |  |
| **HCC** | 355 | 121 | 476 | 74.6% (70.5%-78.3%) |  |
| BCLC 0-A | 279 | 113 | 392 | 71.2% (66.5%-75.4%) |  |
| BCLC 0 | 30 | 35 | 65 | 46.2% (34.6%-58.1%) |  |
| BCLC A | 249 | 78 | 327 | 76.1% (71.2%-80.4%) |  |
| BCLC B-C | 76 | 8 | 84 | 90.5% (82.3%-95.1%) |  |
| **Control** | 145 | 1,071 | 1,216 |  | 88.1% (86.1%-89.8%) |
| Cirrhosis | 76 | 367 | 443 |  | 82.8% (79.1%-86.1%) |
| HBV Hepatitis | 40 | 436 | 476 |  | 91.6% (88.8%-93.8%) |
| Non-HBV Hepatitis | 15 | 154 | 169 |  | 91.1% (85.9%-94.5%) |
| ND | 14 | 114 | 128 |  | 89.1% (82.5%-93.4%) |
| **AFP (cutoff: 20 ng/mL)** |  |  |  |  |  |
| **HCC** | 241 | 235 | 476 | 50.6% (46.2%-55.1%) |  |
| BCLC 0-A | 178 | 214 | 392 | 45.4% (40.5%-50.4%) |  |
| BCLC 0 | 22 | 43 | 65 | 33.8% (23.5%-46%) |  |
| BCLC A | 156 | 171 | 327 | 47.7% (42.4%-53.1%) |  |
| BCLC B-C | 63 | 21 | 84 | 75% (64.8%-83%) |  |
| **Control** | 51 | 1,165 | 1,216 |  | 95.8% (94.5%-96.8%) |
| Cirrhosis | 21 | 422 | 443 |  | 95.3% (92.9%-96.9%) |
| HBV Hepatitis | 24 | 452 | 476 |  | 95% (92.6%-96.6%) |
| Non-HBV Hepatitis | 3 | 166 | 169 |  | 98.2% (94.9%-99.4%) |
| ND | 3 | 125 | 128 |  | 97.7% (93.3%-99.2%) |
| **AFP-L3 (cutoff: 10%)** |  |  |  |  |  |
| **HCC** | 180 | 296 | 476 | 37.8% (33.6%-42.3%) |  |
| BCLC 0-A | 124 | 268 | 392 | 31.6% (27.2%-36.4%) |  |
| BCLC 0 | 13 | 52 | 65 | 20% (12.1%-31.3%) |  |
| BCLC A | 111 | 216 | 327 | 33.9% (29%-39.2%) |  |
| BCLC B-C | 56 | 28 | 84 | 66.7% (56.1%-75.8%) |  |
| **Control** | 70 | 1,146 | 1,216 |  | 94.2% (92.8%-95.4%) |
| Cirrhosis | 26 | 417 | 443 |  | 94.1% (91.5%-96%) |
| HBV Hepatitis | 34 | 442 | 476 |  | 92.9% (90.2%-94.8%) |
| Non-HBV Hepatitis | 7 | 162 | 169 |  | 95.9% (91.7%-98%) |
| ND | 3 | 125 | 128 |  | 97.7% (93.3%-99.2%) |
| **DCP (cutoff: 40 ng/mL)** |  |  |  |  |  |
| **HCC** | 254 | 222 | 476 | 53.4% (48.9%-57.8%) |  |
| BCLC 0-A | 185 | 207 | 392 | 47.2% (42.3%-52.1%) |  |
| BCLC 0 | 13 | 52 | 65 | 20% (12.1%-31.3%) |  |
| BCLC A | 172 | 155 | 327 | 52.6% (47.2%-57.9%) |  |
| BCLC B-C | 69 | 15 | 84 | 82.1% (72.6%-88.9%) |  |
| **Control** | 29 | 1,187 | 1,216 |  | 97.6% (96.6%-98.3%) |
| Cirrhosis | 14 | 429 | 443 |  | 96.8% (94.8%-98.1%) |
| HBV Hepatitis | 7 | 469 | 476 |  | 98.5% (97%-99.3%) |
| Non-HBV Hepatitis | 5 | 164 | 169 |  | 97% (93.3%-98.7%) |
| ND | 3 | 125 | 128 |  | 97.7% (93.3%-99.2%) |

AFP: alpha-fetoprotein; AFP-L3: Lens culinaris agglutinin A-reactive fraction of alpha-fetoprotein; DCP: Des-γ-carboxy-prothrombin; HBV: hepatitis B virus; HCV: hepatitis C virus; BCLC: Barcelona Clinic Liver Cancer; ND; patients with no detectable liver abnormalities.

**Table 6.** **Performance of HepaAiQ in serum tumor marker detection samples.**

| **Sample type** | **Positive** | **Negative** | **Total** | **Sensitivity (95% CI)** | **Specificity (95% CI)** | **Sample type** | **Positive** | **Negative** | **Total** | **Sensitivity (95% CI)** | **Specificity (95% CI)** |
| --- | --- | --- | --- | --- | --- | --- | --- | --- | --- | --- | --- |
| **AFP positive** |  |  |  |  |  | **AFP negative** |  |  |  |  |  |
| **HCC** | 201 | 40 | 241 | 83.4% (78.2%-87.6%) |  | **HCC** | 154 | 81 | 235 | 65.5% (59.2%-71.3%) |  |
| BCLC 0-A | 140 | 38 |  | 78.7% (72.1%-84%) |  | BCLC 0-A | 139 | 75 | 214 | 65% (58.3%-71%) |  |
| BCLC 0 | 10 | 12 | 22 | 45.5% (26.9%-65.3%) |  | BCLC 0 | 20 | 23 | 43 | 46.5% (32.5%-61.1%) |  |
| BCLC A | 130 | 26 | 156 | 83.3% (76.7%-88.4%) |  | BCLC A | 119 | 52 | 171 | 69.6% (62.3%-76%) |  |
| BCLC B-C | 61 | 2 | 63 | 96.8% (89.1%-99.1%) |  | BCLC B-C | 15 | 6 | 21 | 71.4% (50%-86.2%) |  |
| **Control** | 13 | 38 | 51 |  | 74.5% (61.1%-84.5%) | **Control** | 132 | 1,033 | 1,165 |  | 88.7% (86.7%-90.4%) |
| **DCP positive** |  |  |  |  |  | **DCP negative** |  |  |  |  |  |
| **HCC** | 224 | 30 | 254 | 88.2% (83.6%-91.6%) |  | **HCC** | 131 | 91 | 222 | 59% (52.4%-65.3%) |  |
| BCLC 0-A | 156 | 29 | 185 | 84.3% (78.4%-88.9%) |  | BCLC 0-A | 123 | 84 | 207 | 59.4% (52.6%-65.9%) |  |
| BCLC 0 | 4 | 9 | 13 | 30.8% (12.7%-57.6%) |  | BCLC 0 | 26 | 26 | 52 | 50% (36.9%-63.1%) |  |
| BCLC A | 152 | 20 | 172 | 88.4% (82.7%-92.3%) |  | BCLC A | 97 | 58 | 155 | 62.6% (54.7%-69.8%) |  |
| BCLC B-C | 68 | 1 | 69 | 98.6% (92.2%-99.7%) |  | BCLC B-C | 8 | 7 | 15 | 53.3% (30.1%-75.2%) |  |
| **Control** | 11 | 18 | 29 |  | 62.1% (44%-77.3%) | **Control** | 134 | 1,053 | 1,187 |  | 88.7% (86.8%-90.4%) |
| **AFP-L3 positive** |  |  |  |  |  | **AFP-L3 negative** |  |  |  |  |  |
| **HCC** | 155 | 25 | 180 | 86.1% (80.3%-90.4%) |  | **HCC** | 200 | 96 | 296 | 67.6% (62%-72.6%) |  |
| BCLC 0-A | 100 | 24 | 124 | 80.6% (72.8%-86.6%) |  | BCLC 0-A | 179 | 89 | 268 | 66.8% (61%-72.2%) |  |
| BCLC 0 | 5 | 8 | 13 | 38.5% (17.7%-64.5%) |  | BCLC 0 | 25 | 27 | 52 | 48.1% (35.1%-61.3%) |  |
| BCLC A | 95 | 16 | 111 | 85.6% (77.9%-90.9%) |  | BCLC A | 154 | 62 | 216 | 71.3% (64.9%-76.9%) |  |
| BCLC B-C | 55 | 1 | 56 | 98.2% (90.6%-99.7%) |  | BCLC B-C | 21 | 7 | 28 | 75% (56.6%-87.3%) |  |
| **Control** | 14 | 56 | 70 |  | 80% (69.2%-87.7%) | **Control** | 131 | 1,015 | 1,146 |  | 88.6% (86.6%-90.3%) |
| **AFP or DCP positive** |  |  |  |  |  | **AFP and DCP negative** |  |  |  |  |  |
| **HCC** | 269 | 54 | 323 | 83.3% (78.8%-87%) |  | **HCC** | 86 | 67 | 153 | 56.2% (48.3%-63.8%) |  |
| BCLC 0-A | 199 | 52 | 251 | 79.3% (73.8%-83.8%) |  | BCLC 0-A | 80 | 61 | 141 | 56.7% (48.5%-64.6%) |  |
| BCLC 0 | 12 | 15 | 27 | 44.4% (27.6%-62.7%) |  | BCLC 0 | 18 | 20 | 38 | 47.4% (32.5%-62.7%) |  |
| BCLC A | 187 | 37 | 224 | 83.5% (78.1%-87.8%) |  | BCLC A | 62 | 41 | 103 | 60.2% (50.5%-69.1%) |  |
| BCLC B-C | 70 | 2 | 72 | 97.2% (90.4%-99.2%) |  | BCLC B-C | 6 | 6 | 12 | 50% (25.4%-74.6%) |  |
| **Control** | 22 | 52 | 74 |  | 70.3% (59.1%-79.5%) | **Control** | 123 | 1,019 | 1,142 |  | 89.2% (87.3%-90.9%) |
| **AFP or DCP or AFP-L3 positive** |  |  |  |  |  | **AFP and DCP and AFP-L3 negative** |  |  |  |  |  |
| **HCC** | 269 | 54 | 323 | 83.3% (78.8%-87%) |  | **HCC** | 86 | 67 | 153 | 56.2% (48.3%-63.8%) |  |
| BCLC 0-A | 199 | 52 | 251 | 79.3% (73.8%-83.8%) |  | BCLC 0-A | 80 | 61 | 141 | 56.7% (48.5%-64.6%) |  |
| BCLC 0 | 12 | 15 | 27 | 44.4% (27.6%-62.7%) |  | BCLC 0 | 18 | 20 | 38 | 47.4% (32.5%-62.7%) |  |
| BCLC A | 187 | 37 | 224 | 83.5% (78.1%-87.8%) |  | BCLC A | 62 | 41 | 103 | 60.2% (50.5%-69.1%) |  |
| BCLC B-C | 70 | 2 | 72 | 97.2% (90.4%-99.2%) |  | BCLC B-C | 6 | 6 | 12 | 50% (25.4%-74.6%) |  |
| **Control** | 28 | 75 | 103 |  | 72.8% (63.5%-80.5%) | **Control** | 117 | 996 | 1,113 |  | 89.5% (87.5%-91.2%) |

Abbreviation: AFP: alpha-fetoprotein; AFP-L3: Lens culinaris agglutinin A-reactive fraction of alpha-fetoprotein; DCP: Des-γ-carboxy-prothrombin; HBV: hepatitis B virus; HCV: hepatitis C virus; BCLC: Barcelona Clinic Liver Cancer; CI: confidence interval.

**Table 7. OR (95% CI) of variables in all samples.**

|  | OR (univariable) | OR (multivariable) |
| --- | --- | --- |
| Gender (Male VS. Female) | 2.78 (2.10-3.69, p < .001) | 3.00 (1.95-4.61, p < .001) |
| Age | 1.08 (1.07-1.09, p < .001) | 1.08 (1.06-1.10, p < .001) |
| ln AFP | 2.15 (1.95-2.38, p < .001) | 1.63 (1.37-1.94, p < .001) |
| AFP_L3 | 1.24 (1.19-1.30, p < .001) | 1.03 (0.97-1.09, p = .336) |
| ln DCP | 2.81 (2.48-3.18, p < .001) | 1.72 (1.48-2.00, p < .001) |
| HepaAiQ score | 8.51 (6.52-11.11, p < .001) | 4.32 (3.24-5.75, p < .001) |

Abbreviation: CI: confidence interval; AFP: alpha-fetoprotein; AFP-L3: Lens culinaris agglutinin A-reactive fraction of alpha-fetoprotein; DCP: Des-γ-carboxy-prothrombin. Wald test.

**Table 8. Diagnostic performances of GALAD in training, validation, and test cohort.**

| **Sample type** | **Positive** | **Negative** | **Total** | **Sensitivity (95% CI)** | **Specificity (95% CI)** |
| --- | --- | --- | --- | --- | --- |
| **Training** |  |  |  |  |  |
| **HCC** | 128 | 31 | 159 | 80.5% (73.7%-85.9%) |  |
| BCLC 0-A | 128 | 31 | 159 | 80.5% (73.7%-85.9%) |  |
| BCLC 0 | 17 | 9 | 26 | 65.4% (46.2%-80.6%) |  |
| BCLC A | 111 | 22 | 133 | 83.5% (76.2%-88.8%) |  |
| BCLC B-C | 0 | 0 | 0 | 83.5% (76.2%-88.8%) |  |
| **Control** | 65 | 339 | 404 |  | 83.9% (80%-87.2%) |
| Cirrhosis | 32 | 115 | 147 |  | 78.2% (70.9%-84.1%) |
| HBV Hepatitis | 22 | 136 | 158 |  | 86.1% (79.8%-90.6%) |
| Non-HBV Hepatitis | 9 | 47 | 56 |  | 83.9% (72.2%-91.3%) |
| ND | 2 | 41 | 43 |  | 95.3% (84.5%-98.7%) |
| **Validation** |  |  |  |  |  |
| **HCC** | 129 | 30 | 159 | 81.1% (74.3%-86.5%) |  |
| BCLC 0-A | 129 | 30 | 159 | 81.1% (74.3%-86.5%) |  |
| BCLC 0 | 19 | 8 | 27 | 70.4% (51.5%-84.1%) |  |
| BCLC A | 110 | 22 | 132 | 83.3% (76.1%-88.7%) |  |
| BCLC B-C | 0 | 0 | 0 | 83.3% (76.1%-88.7%) |  |
| **Control** | 80 | 325 | 405 |  | 80.2% (76.1%-83.8%) |
| Cirrhosis | 41 | 107 | 148 |  | 72.3% (64.6%-78.9%) |
| HBV Hepatitis | 21 | 138 | 159 |  | 86.8% (80.7%-91.2%) |
| Non-HBV Hepatitis | 12 | 44 | 56 |  | 78.6% (66.2%-87.3%) |
| ND | 6 | 36 | 42 |  | 85.7% (72.2%-93.3%) |
| **Test** |  |  |  |  |  |
| **HCC** | 137 | 21 | 158 | 86.7% (80.5%-91.1%) |  |
| BCLC 0-A | 60 | 14 | 74 | 81.1% (70.7%-88.4%) |  |
| BCLC 0 | 8 | 4 | 12 | 66.7% (39.1%-86.2%) |  |
| BCLC A | 52 | 10 | 62 | 83.9% (72.8%-91%) |  |
| BCLC B-C | 77 | 7 | 84 | 91.7% (83.8%-95.9%) |  |
| **Control** | 63 | 344 | 407 |  | 84.5% (80.7%-87.7%) |
| Cirrhosis | 35 | 113 | 148 |  | 76.4% (68.9%-82.5%) |
| HBV Hepatitis | 20 | 139 | 159 |  | 87.4% (81.4%-91.7%) |
| Non-HBV Hepatitis | 5 | 52 | 57 |  | 91.2% (81.1%-96.2%) |
| ND | 3 | 40 | 43 |  | 93% (81.4%-97.6%) |
| **Total** |  |  |  |  |  |
| **HCC** | 394 | 82 | 476 | 82.8% (79.1%-85.9%) |  |
| BCLC 0-A | 317 | 75 | 392 | 80.9% (76.7%-84.5%) |  |
| BCLC 0 | 44 | 21 | 65 | 67.7% (55.6%-77.8%) |  |
| BCLC A | 273 | 54 | 327 | 83.5% (79.1%-87.1%) |  |
| BCLC B-C | 77 | 7 | 84 | 91.7% (83.8%-95.9%) |  |
| **Control** | 208 | 1,008 | 1,216 |  | 82.9% (80.7%-84.9%) |
| Cirrhosis | 108 | 335 | 443 |  | 75.6% (71.4%-79.4%) |
| HBV Hepatitis | 63 | 413 | 476 |  | 86.8% (83.4%-89.5%) |
| Non-HBV Hepatitis | 26 | 143 | 169 |  | 84.6% (78.4%-89.3%) |
| ND | 11 | 117 | 128 |  | 91.4% (85.3%-95.1%) |

Abbreviation: HBV: hepatitis B virus; HCV: hepatitis C virus; ND; patients with no detectable liver abnormalities; CI: confidence interval; BCLC: Barcelona Clinic Liver Cancer.

**Table 9. Performance of GAMAD in GALAD assessed samples.**

| **Sample type** | **Positive** | **Negative** | **Total** | **Sensitivity (95% CI)** | **Specificity (95% CI)** |
| --- | --- | --- | --- | --- | --- |
| **GALAD positive** |  |  |  |  |  |
| **HCC** | 373 | 21 | 394 | 94.7% (92%-96.5%) |  |
| BCLC 0-A | 297 | 20 | 317 | 93.7% (90.5%-95.9%) |  |
| BCLC 0 | 37 | 7 | 44 | 84.1% (70.6%-92.1%) |  |
| BCLC A | 260 | 13 | 273 | 95.2% (92%-97.2%) |  |
| BCLC B-C | 76 | 1 | 77 | 98.7% (93%-99.8%) |  |
| **Control** | 94 | 114 | 208 |  | 54.8% (48%-61.4%) |
| Cirrhosis | 55 | 53 | 108 |  | 49.1% (39.8%-58.4%) |
| HBV Hepatitis | 24 | 39 | 63 |  | 61.9% (49.6%-72.9%) |
| Non-HBV Hepatitis | 11 | 15 | 26 |  | 57.7% (38.9%-74.5%) |
| ND | 4 | 7 | 11 |  | 63.6% (35.4%-84.8%) |
| **GALAD negative** |  |  |  |  |  |
| **HCC** | 33 | 49 | 82 | 40.2% (30.3%-51.1%) |  |
| BCLC 0-A | 32 | 43 | 75 | 42.7% (32.1%-53.9%) |  |
| BCLC 0 | 6 | 15 | 21 | 28.6% (13.8%-50%) |  |
| BCLC A | 26 | 28 | 54 | 48.1% (35.4%-61.1%) |  |
| BCLC B-C | 1 | 6 | 7 | 14.3% (2.57%-51.3%) |  |
| **Control** | 23 | 985 | 1,008 |  | 97.7% (96.6%-98.5%) |
| Cirrhosis | 14 | 321 | 335 |  | 95.8% (93.1%-97.5%) |
| HBV Hepatitis | 5 | 408 | 413 |  | 98.8% (97.2%-99.5%) |
| Non-HBV Hepatitis | 2 | 141 | 143 |  | 98.6% (95%-99.6%) |
| ND | 2 | 115 | 117 |  | 98.3% (94%-99.5%) |

Abbreviation: CI: confidence interval; HBV: hepatitis B virus; HCV: hepatitis C virus; BCLC: Barcelona Clinic Liver Cancer; ND; patients with no detectable liver abnormalities.

**Table 10. Diagnostic performances of the individual marker, GAMAD, and GALAD in cirrhosis subgroup.**

| **Sample type** | **Sensitivity**  (n/n) | **Specificity**  (n/n) | **Sensitivity**  (n/n) | **Specificity**  (n/n) | **Sensitivity**  (n/n) | **Specificity**  (n/n) |
| --- | --- | --- | --- | --- | --- | --- |
|  | **HepaAiQ (Positive/Negative)** | | **AFP (cutoff: 20ng/mL)** | | **AFP-L3 (cutoff: 10%)** | |
| **HCC** | 81.4% (275/338) |  | 58.0% (196/338) |  | 44.7% (151/338) |  |
| BCLC 0-A | 79.2% (213/269) |  | 52.0% (140/269) |  | 37.9% (102/269) |  |
| BCLC 0 | 65.7% (23/35) |  | 28.6% (10/35) |  | 17.1% (6/35) |  |
| BCLC A | 81.2% (190/234) |  | 55.6% (130/234) |  | 41.0% (96/234) |  |
| BCLC B-C | 89.9% (62/69) |  |  |  | 71.0% (49/69) |  |
| **Control** |  | 82.8% (367/443) |  | 95.3% (422/443) |  | 94.1% (417/443) |
| Cirrhosis |  | 82.8% (367/443) |  | 95.3% (422/443) |  | 94.1% (417/443) |
|  | **DCP (cutoff: 40ng/mL)** | | **GALAD (Positive/Negative)** | | **GAMAD (Positive/Negative)** | |
| **HCC** | 60.9% (206/338) |  | 86.1% (291/338) |  | 89.3% (302/338) |  |
| BCLC 0-A | 54.3% (146/269) |  | 83.6% (225/269) |  | 88.1% (237/269) |  |
| BCLC 0 | 20.0% (7/35) |  | 62.9% (22/35) |  | 71.4% (25/35) |  |
| BCLC A | 59.4% (139/234) |  | 86.8% (203/234) |  | 90.6% (212/234) |  |
| BCLC B-C | 87.0% (60/69) |  | 95.7% (66/69) |  | 94.2% (65/69) |  |
| **Control** |  | 96.8% (429/443) |  | 75.6% (335/443) |  | 84.4% (374/443) |
| Cirrhosis |  | 96.8% (429/443) |  | 75.6% (335/443) |  | 84.4% (374/443) |

Abbreviation: AFP: alpha-fetoprotein; AFP-L3: Lens culinaris agglutinin A-reactive fraction of alpha-fetoprotein; DCP: Des-γ-carboxy-prothrombin. BCLC: Barcelona Clinic Liver Cancer.

**Table 11. Diagnostic performances of the individual marker, GAMAD, and GALAD in HBV subgroup.**

| **Sample type** | **Sensitivity**  (n/n) | **Specificity**  (n/n) | **Sensitivity**  (n/n) | **Specificity**  (n/n) | **Sensitivity**  (n/n) | **Specificity**  (n/n) |
| --- | --- | --- | --- | --- | --- | --- |
|  | **HepaAiQ (Positive/Negative)** | | **AFP (cutoff: 20ng/mL)** | | **AFP-L3 (cutoff: 10%)** | |
| **HCC** | 73.9% (281/380) |  | 52.4% (199/380) |  | 38.4% (146/380) |  |
| BCLC 0-A | 71.5% (231/323) |  | 48.3% (156/323) |  | 33.7% (109/323) |  |
| BCLC 0 | 48.0% (24/50) |  | 36.0% (18/50) |  | 22.0% (11/50) |  |
| BCLC A | 75.8% (207/273) |  | 50.5% (138/273) |  | 35.9% (98/273) |  |
| BCLC B-C | 87.7% (50/57) |  | 75.4% (43/57) |  | 64.9% (37/57) |  |
| **Control** |  | 88.5% (699/790) |  | 94.7% (748/790) |  | 93.5% (739/790) |
| Cirrhosis |  | 83.8% (263/314) |  | 94.3% (296/314) |  | 94.6% (297/314) |
| HBV Hepatitis |  | 91.6% (436/476) |  | 95.0% (452/476) |  | 92.9% (442/476) |
|  | **DCP (cutoff: 40ng/mL)** | | **GALAD (Positive/Negative)** | | **GAMAD (Positive/Negative)** | |
| **HCC** | 55.0% (209/380) |  | 82.4% (313/380) |  | 86.1% (327/380) |  |
| BCLC 0-A | 49.8% (161/323) |  | 80.5% (260/323) |  | 85.1% (275/323) |  |
| BCLC 0 | 18.0% (9/50) |  | 66.0% (33/50) |  | 68.0% (34/50) |  |
| BCLC A | 55.7% (152/273) |  | 83.2% (227/273) |  | 88.3% (241/273) |  |
| BCLC B-C | 84.2% (48/57) |  | 93.0% (53/57) |  | 91.2% (52/57) |  |
| **Control** |  | 98.6% (779/790) |  | 82.7% (653/790) |  | 90.9% (718/790) |
| Cirrhosis |  | 98.7% (310/314) |  | 76.4% (240/314) |  | 86.3% (271/314) |
| HBV Hepatitis |  | 98.5% (469/476) |  | 86.8% (413/476) |  | 93.9% (447/476) |

Abbreviation: AFP: alpha-fetoprotein; AFP-L3: Lens culinaris agglutinin A-reactive fraction of alpha-fetoprotein; DCP: Des-γ-carboxy-prothrombin. BCLC: Barcelona Clinic Liver Cancer.

**Table 12. Diagnostic performances of the individual marker, GAMAD, and GALAD in non-HBV subgroup.**

| Sample type | Sensitivity  (n/n) | Specificity  (n/n) | Sensitivity  (n/n) | Specificity  (n/n) | Sensitivity  (n/n) | Specificity  (n/n) |
| --- | --- | --- | --- | --- | --- | --- |
|  | HepaAiQ (Positive/Negative) | | AFP (cutoff: 20ng/mL) | | AFP-L3 (cutoff: 10%) | |
| HCC | 77.1% (74/96) |  | 43.8% (42/96) |  | 35.4% (34/96) |  |
| BCLC 0-A | 69.6% (48/69) |  | 31.9% (22/69) |  | 21.7% (15/69) |  |
| BCLC 0 | 40.0% (6/15) |  | 26.7% (4/15) |  | 13.3% (2/15) |  |
| BCLC A | 77.8% (42/54) |  | 33.3% (18/54) |  | 24.1% (13/54) |  |
| BCLC B-C | 96.3% (26/27) |  | 74.1% (20/27) |  | 70.4% (19/27) |  |
| Control |  | 87.3% (372/426) |  | 97.9% (417/426) |  | 95.5% (407/426) |
| Cirrhosis |  | 80.6% (104/129) |  | 97.7% (126/129) |  | 93.0% (120/129) |
| Non HBV Hepatitis |  | 91.1% (154/169) |  | 98.2% (166/169) |  | 95.9% (162/169) |
| ND |  | 89.1% (114/128) |  | 97.7% (125/128) |  | 97.7% (125/128) |
|  | DCP (cutoff: 40ng/mL) | | GALAD (Positive/Negative) | | GAMAD (Positive/Negative) | |
| HCC | 46.9% (45/96) |  | 84.4% (81/96) |  | 82.3% (79/96) |  |
| BCLC 0-A | 34.8% (24/69) |  | 82.6% (57/69) |  | 78.3% (54/69) |  |
| BCLC 0 | 26.7% (4/15) |  | 73.3% (11/15) |  | 60.0% (9/15) |  |
| BCLC A | 37.0% (20/54) |  | 85.2% (46/54) |  | 83.3% (45/54) |  |
| BCLC B-C | 77.8% (21/27) |  | 88.9% (24/27) |  | 92.6% (25/27) |  |
| Control |  | 95.8% (408/426) |  | 83.3% (355/426) |  | 89.4% (381/426) |
| Cirrhosis |  | 92.2% (119/129) |  | 73.6% (95/129) |  | 79.8% (103/129) |
| HBV Hepatitis |  | 97.0% (164/169) |  | 84.6% (143/169) |  | 92.3% (156/169) |
| ND |  | 97.7% (125/128) |  | 91.4% (117/128) |  | 95.3% (122/128) |

Abbreviation: AFP: alpha-fetoprotein; AFP-L3: Lens culinaris agglutinin A-reactive fraction of alpha-fetoprotein; DCP: Des-γ-carboxy-prothrombin. BCLC: Barcelona Clinic Liver Cancer.

**Table 13. Sensitivities (%) of the GAMAD and GALAD models and individual biomarkers at the fixed specificity of 90% in all samples.**

|  | All stage | |  | BCLC 0/A |  |  | BCLC 0 |  |  |
| --- | --- | --- | --- | --- | --- | --- | --- | --- | --- |
| Model and markers | Cutoff | Sensitivity | P value | Cutoff | Sensitivity | P value | Cutoff | Sensitivity | P value |
| GAMAD (Logit) | -1.084 | 85.5% | Reference | -1.084 | 83.9% | Reference | -1.084 | 66.2% | Reference |
| GALAD | -0.017 | 76.7% | 0.001 | -0.017 | 73.7% | < 0.001 | -0.017 | 53.8% | 0.152 |
| HepaAiQ score | -0.947 | 72.5% | < 0.001 | -0.947 | 68.9% | < 0.001 | -0.947 | 43.1% | 0.008 |
| AFP (ng/mL) | 8.435 | 59.9% | < 0.001 | 8.435 | 54.8% | < 0.001 | 8.435 | 47.7% | 0.034 |
| AFP-L3 (%) | 5.050 | 47.9% | < 0.001 | 5.050 | 41.6% | < 0.001 | 5.350 | 30.8% | < 0.001 |
| DCP (ng/mL) | 13.675 | 67.4% | < 0.001 | 13.675 | 63.0% | < 0.001 | 13.675 | 30.8% | < 0.001 |

Abbreviation: AFP: alpha-fetoprotein; AFP-L3: Lens culinaris agglutinin A-reactive fraction of alpha-fetoprotein; DCP: Des-γ-carboxy-prothrombin. Chi-square test.

**Table 14. Sensitivities (%) of the GAMAD and GALAD models and individual biomarkers at the fixed specificity of 90% in the cirrhosis subgroup.**

|  | All stage | |  | BCLC 0/A |  |  | BCLC 0 |  |  |
| --- | --- | --- | --- | --- | --- | --- | --- | --- | --- |
| Model and markers | Cutoff | Sensitivity | P value | Cutoff | Sensitivity | P value | Cutoff | Sensitivity | P value |
| GAMAD (Logit) | -0.362 | 82.5% | Reference | -0.362 | 80.7% | Reference | -0.352 | 54.3% | Reference |
| GALAD | 0.409 | 75.1% | 0.019 | 0.409 | 70.6% | 0.007 | 0.433 | 40.0% | 0.231 |
| HepaAiQ score | -0.752 | 72.5% | 0.002 | -0.752 | 70.3% | 0.005 | -0.752 | 40.0% | 0.231 |
| AFP (ng/mL) | 10.405 | 63.3% | < 0.001 | 10.405 | 57.6% | < 0.001 | 10.405 | 37.1% | 0.150 |
| AFP-L3 (%) | 5.050 | 54.1% | < 0.001 | 5.050 | 46.8% | < 0.001 | 5.500 | 28.6% | 0.029 |
| DCP (ng/mL) | 16.270 | 73.7% | 0.004 | 16.270 | 69.1% | 0.002 | 16.270 | 31.4% | 0.053 |

Abbreviation: AFP: alpha-fetoprotein; AFP-L3: Lens culinaris agglutinin A-reactive fraction of alpha-fetoprotein; DCP: Des-γ-carboxy-prothrombin. BCLC: Barcelona Clinic Liver Cancer. Chi-square test.

**Table 15. Sensitivities (%) of the GAMAD and GALAD models and individual biomarkers at the fixed specificity of 90% in the HBV group.**

|  | All stage | |  | BCLC 0/A |  |  | BCLC 0 |  |  |
| --- | --- | --- | --- | --- | --- | --- | --- | --- | --- |
| Model and markers | Cutoff | Sensitivity | P value | Cutoff | Sensitivity | P value | Cutoff | Sensitivity | P value |
| GAMAD (Logit) | -1.143 | 86.3% | Reference | -1.143 | 85.4% | Reference | -1.137 | 68.0% | Reference |
| GALAD | 0.022 | 76.6% | 0.001 | 0.022 | 74.0% | < 0.001 | 0.032 | 54.0% | 0.151 |
| HepaAiQ score | -0.988 | 72.9% | < 0.001 | -0.988 | 70.3% | < 0.001 | -0.987 | 46.0% | 0.026 |
| AFP (ng/mL) | 9.110 | 59.2% | < 0.001 | 9.110 | 55.1% | < 0.001 | 9.110 | 46.0% | 0.026 |
| AFP-L3 (%) | 5.050 | 47.9% | < 0.001 | 5.050 | 43.0% | < 0.001 | 5.500 | 30.0% | < 0.001 |
| DCP (ng/mL) | 13.530 | 69.5% | < 0.001 | 13.530 | 65.3% | < 0.001 | 13.620 | 30.0% | < 0.001 |

Abbreviation: AFP: alpha-fetoprotein; AFP-L3: Lens culinaris agglutinin A-reactive fraction of alpha-fetoprotein; DCP: Des-γ-carboxy-prothrombin. BCLC: Barcelona Clinic Liver Cancer. Chi-square test.

**Table 16. Comparison of reclassification performance between GAMAD and GALAD models: Categorical and Continuous Net Reclassification Improvement (NRI) and Integrated Discrimination Improvement (IDI).**

|  | NRI(Categorical) (95% CI) | P value | NRI(Continuous) (95% CI) | P value | IDI | P value | AUDCA |
| --- | --- | --- | --- | --- | --- | --- | --- |
| GALAD | Ref |  | Ref |  | Ref |  | 0.132 |
| GAMAD | 0.07 (0.031 - 0.109) | <0.001 | 0.9592 (0.8642 - 1.0542) | <0.001 | 0.0872 (0.0626 - 0.1119) | <0.001 | 0.157 |

AUDCA: area under the decision curve analysis.

**Table 17. Monte-Carlo simulation to predict early detection of HCC.**

|  | Ultrasound with AFP (95%CI) | GAMAD (95%CI) |
| --- | --- | --- |
| Number of adherences | 39,214 (30,294-49,490) | 75,026 (66,482-83,605) |
| Number of detected early HCC | 1,030 (657-1,519) | 2,630 (1,879-3,563) |
| False negative rate | 37.2% (27.8%-47.1%) | 16.3% (9.5%-24.0%) |
| PPV | 15.1% (9.2%-24.4%) | 28.0% (17.2%-44.3%) |
| NPV | 98.1% (97.3%-98.8%) | 99.2% (98.7%-99.6%) |

Abbreviation: HBV: CI: confidence interval; HCC: hepatocellular carcinoma; PPV: positive predictive value; NPV: negative predictive value.

# **References**

1. Guo DZ, Huang A, Wang YC, Zhou S, Wang H, Xing XL et al. Early detection and prognosis evaluation for hepatocellular carcinoma by circulating tumour DNA methylation: A multicentre cohort study. Clinical and translational medicine. 2024;14(5):e1652. doi:10.1002/ctm2.1652.
